# Gut microbiota distinct between colorectal cancers with deficient and proficient mismatch repair: A study of 230 CRC patients

**DOI:** 10.3389/fmicb.2022.993285

**Published:** 2022-10-13

**Authors:** Min Jin, Jingjing Wu, Linli Shi, Bin Zhou, Fumei Shang, Xiaona Chang, Xiaochuan Dong, Shenghe Deng, Li Liu, Kailin Cai, Xiu Nie, Tao Zhang, Jun Fan, Hongli Liu

**Affiliations:** ^1^Cancer Center, Union Hospital, Tongji Medical College, Huazhong University of Science and Technology, Wuhan, China; ^2^Institute of Radiation Oncology, Union Hospital, Tongji Medical College, Huazhong University of Science and Technology, Wuhan, China; ^3^Department of Radiation Oncology, Cancer Center, The First Affiliated Hospital of Xiamen University, Xiamen, China; ^4^Department of Pathology, Union Hospital, Tongji Medical College, Huazhong University of Science and Technology, Wuhan, China; ^5^Department of Gastrointestinal Surgery, Union Hospital, Tongji Medical College, Huazhong University of Science and Technology, Wuhan, China; ^6^Department of Epidemiology and Biostatistics, The Ministry of Education Key Lab of Environment and Health, School of Public Health, Tongji Medical College, Huazhong University of Science and Technology, Wuhan, China

**Keywords:** colorectal cancer, gut microbiota, proficient DNA mismatch repair, deficient DNA mismatch repair, *Fusobacterium*, *Akkermansia*, *Bifidobacterium*

## Abstract

Colorectal cancers (CRCs) with deficient DNA mismatch repair (dMMR) and proficient DNA mismatch repair (pMMR) exhibit heterogeneous tumor characteristics, distinct responses to immunotherapy, and different survival outcomes. However, it is unclear whether gut microbiota is distinct between CRCs with different MMR status. In this study, we used immunohistochemistry for four major MMR proteins to determine the MMR status in 230 CRC patients. The gut microbiota was profiled in cancerous and adjacent normal tissues by using bacterial 16S rRNA sequencing. The differences in microbiota diversity, composition and related metabolic pathways between patients with dMMR and pMMR CRCs were explored. Linear discriminant analysis effect size (LEfSe) analysis was further applied to validate the significant taxonomic differences at the genus level. In our study cohort, dMMR status was identified in 29 of 230 (12.61%) tumors. The richness (alpha-diversity) of gut microbiome in dMMR tumor tissue was higher compared with pMMR tumor tissues. The microbial community composition (beta-diversity) between the two groups was significantly different. The dMMR group was enriched considerably for some microbiota, including Fusobacteria, Firmicutes, Verrucomicrobia, and Actinobacteria at the phylum level and *Fusobacterium, Akkermansia*, *Bifidobacterium*, *Faecalibacterium*, *Streptococcus*, and *Prevotella* bacteria at the genus level. However, the pMMR group was dominated by Proteobacteria at the phylum level and *Serratia*, *Cupriavidu*s and *Sphingobium* at the genus level. Moreover, a wide variety of microbiota associated functional pathways were observed with different MMR status. KEGG pathway analysis indicated a higher abundance of the biosynthesis and metabolic pathways of glycan and nucleotide, cell growth and death pathways, genetic replication and repair pathways in dMMR samples compared with the pMMR group. These findings demonstrate that CRC patients with different MMR status have distinct gut bacterial community richness, compositions and related metabolic pathways, suggesting basis that may explain the effectiveness of immunotherapy in dMMR tumors.

## Introduction

Colorectal cancer (CRC) is characterized as a multifactorial and heterogeneous disease. CRC is the third most common cancer with a high mortality rate worldwide (Ferlay et al., 2015). At least three pathways have been identified as contributing to CRC development, including chromosomal instability (CIN), CpG island methylator phenotype (CIMP), and microsatellite instability (MSI) (Siddique et al., 2016). MSI is characterized by a high quantity of mutations in microsatellite locations which results from mutations or silencing of genes coding for mismatch repair (MMR) proteins (MLH1, MSH2, MSH6, and PMS2). According to MMR status, CRCs can also be classified into mismatch-repair-deficient (dMMR)/microsatellite instability-high (MSI-H) subtypes and mismatch-repair-proficient (pMMR)/microsatellite stability (MSS) subtypes (Ganesh et al., 2019). Approximately, 15% of sporadic CRCs are dMMR (Richman, 2015). Many studies have reported the various differences between the dMMR and pMMR in terms of clinicopathological characteristics, immunotherapy sensitivity, and prognosis. Compared to pMMR CRCs, dMMR CRCs often occur on the right side of the colon, have a low rate of metastasis and have better prognosis (Ganesh et al., 2019; Xu et al., 2022). Moreover, dMMR CRC also has higher rates of somatic mutations and increases the probability of neoantigen formation resulting in greater immune system activation. The programmed death receptor-1 (PD-1) antagonists such as pembrolizumab were approved by the Food and Drug Administration (FDA) for unresectable or metastatic CRC patients with MSI-H/dMMR, but not pMMR, according to the results of open-label randomized trial of keynote-177 (Casak et al., 2021).

The specific mechanism by which the MMR status affects the pathogenesis of CRC has not been fully elucidated. In the last few years, the role of the microbiome in the development of CRC has been increasingly emphasized. Numerous studies have proven that CRC patients have an altered gut microbiome compared to healthy individuals. For example, an overabundance of the bacterial organisms, including *Fusobacterium nucleatum* (*F. nucleatum*), *Peptostreptococcus anaerobius* and enterotoxigenic *Bacteroides fragilis*, has an important role in the carcinogenesis of CRC, causing inflammation and abnormal signaling pathways (Zhou et al., 2020; Velikova et al., 2021). The dysbiosis of gut microbiota also promotes intestinal barrier permeability, facilitatse bacterial translocation, and macrophage activation, contributing to a pro-tumorigenic inflammatory microenvironment. Several clues have suggested there may be interaction between MMR status and gut microbiota. Firstly, dMMR CRC predicts a strong response to immune-checkpoint inhibitors (ICIs). Further, the efficacy of immunotherapy is increasingly recognized to be linked to the gut microbiome (Wardill et al., 2022). Secondly, CRC with different MMR status harbor different metabolic profiles, while microbiota has been recently found to affect host metabolism (Kindt et al., 2018; Granado-Serrano et al., 2019). Thirdly, according to tumor consensus molecular subtypes (CMS), dMMR CRCs usually belong to CMS 1 (Grabovska et al., 2020), while Rachel V. et al. found that certain gut microbiome patterns including *Fusobacterium hwasookii* and *Porphyromonas gingivalis* enrichment is associated with the CMS 1 subtype in CRC (Purcell et al., 2017). Nonetheless, evidence demonstrating a direct correlation between MMR status and microbiome composition is generally lacking, particularly in Asian populations. The interaction needs to be investigated in depth to obtain further insights into the mechanisms involved in the tumorigenesis of CRC with different MMR status.

In this study, to investigate the differences in the gut microbiome between CRCs varying MMR status, we collected 230 CRC tumor tissues and the matched normal-adjacent tissue samples. The tumor MMR states were estimated *via* analysis the expression levels of the four most common MMR proteins. The CRC patients were classified into dMMR or pMMR groups. We then evaluated and compared the richness and composition of the gut microbiome in samples between different MMR status and between tumors tissues and normal-adjacent tissue samples using the next generation sequencing (NGS) of 16S ribosomal RNA (rRNA).

## Materials and methods

### Subject enrollment and sample preparation

This study enrolled 230 CRC patients receiving partial or total colectomy from January 01, 2015 to December 31, 2017 at the Union Hospital of Tongji Medical College, Huazhong University of Science and Technology. The inclusion criteria were (1) age > 18 years with a histological diagnosis of primary CRC; (2) received surgical treatment; (3) with the available tumor and adjacent normal tissues. Patients who had received chemotherapy, radiotherapy, or antibiotics in the 2 weeks preceding surgery were excluded from this study. Samples obtained from the caecum to the splenic flexure were defined as proximal, while those obtained from the descending colon to the rectum were defined as distal.

Samples of paired tumor and normal mucosa tissue were fixed in formalin and embedded in paraffin (FFPE). Five serial cuts of 5 μM per sample were placed in sterile containers and kept at room temperature for immunohistochemistry (IHC) staining and 16S rRNA MiSeq sequencing. MMR status was determined by four major MMR protein (MLH1, MSH2, MSH6, and PMS2) expressions in FFPE tissue samples *via* IHC staining. Loss of one or more MMR proteins was classified as dMMR. The converse was defined as pMMR. Tumor and normal paracancerous samples from dMMR CRC patients were designated as dMMR-T and dMMR-N, respectively, while tumor and normal paracancerous samples from pMMR CRC patients were designated as pMMR-T and pMMR-N. The study was conducted according to the guidelines of the Declaration of Helsinki, and approved by the Ethics Committee of Tongji Medical College of Huazhong University of Science and Technology (No. 2021–0793). Written informed consent was obtained from all subjects involved in the study.

### DNA extraction, PCR amplification, and MiSeq sequencing

Bacterial genomic DNA was extracted from CRC tissues and paired normal mucosal tissues using the Omega Mag-Bind Soil DNA Kit (M5635-02) (Omega Bio-Tek, Norcross, GA, United States). DNA quantification and quality were performed by ultraviolet spectrophotometry (Thermo Fisher Scientific, NC2000), and its size was confirmed by 0.8% agarose gel electrophoresis. All extracted DNA samples were immediately maintained at-80°C. The extracted DNA was amplified by employing primers targeting the V3-V4 hypervariable16S rRNA region. The forward primer was 5′-ACTCCTACGGGAGGCAGCA-3′ and the reverse primer was 5′-GGACTACHVGGGTWTCTAAT-3′.PCR was conducted in ABI 2720 under the following conditions: initial denaturation at 98°C for 2 min; 30 cycles of 15 s at 98°C, 30 s at 55°C, and 30 s at 72°C; with a final extension at 72°C for 5 min. The correct PCR products were verified using 2% agarose gel electrophoresis. After gel purification, the amplicons were quantified by the Quant-iTPicoGreen dsDNA Assay Kit on Microplate reader (BioTek, FLx800). The amplicon libraries were then constructed using the TruSeq Nano DNA LT Library Prep Kit. The final libraries were normalized and pooled for a 2 × 300 BP double-ended sequencing on Illumina MiSeq using MiSeq Reagent Kit V3 (600 cycles).

### Sequencing data processing

The obtained sequences were merged and divided into Operational Taxonomic Units (OTUs) used for phylogenetic analysis and taxonomic identification. The diversity of per sample was assessed by the abundance of OTUs in distinct samples.

#### Original data’s collation, filtration, and quality assessment

In order, QIIME17 (v1.8.0) was used to filter possible errors or doubtful sequences in high-throughput sequencing. Then we accepted the sequences with length greater than 160 bp, and the existence of ambiguous bases were not allowed. The following sequences were excluded: (1) sequences with 5′end primer mismatched base number > 1; (2) sequences with continuous same base number > 8. Then USEARCH (v5.2.236) was processed using QIIME software (v1.8.0) to check and delete the chimeric sequences.

#### OTU taxonomy and identification of taxonomic status

The USEARCH software (v5.2.236) was used to cluster and classify the qualified sequences with 97% similarity using the UPARSE-OTU algorithm and screen representative sequences in each OUT with the most abundant sequences. Greengenes database (Release 13.8) was selected as 16S rRNA gene databases for bacteria and archaea. According to the OTU abundance matrix, the proportions of common and unique OTUs per sample was visually displayed by the Venn diagram based on consensus OTUs of each sample calculated by R software.

#### Analysis of alpha-and beta-diversity

Alpha-diversity is a comprehensive indicator reflecting community richness and species evenness within samples *via* four indices, including the ACE, Chao1, Simpson, and Shannon indices. The index in the community was performed by QIIME and R software. Beta-diversity analysis was utilized to estimate the differences of species complexity on both the unweighted and weighted unifrac and visualize the species displayed by Principal coordinate analysis (PCoA) using QIIME and R software.

#### Taxonomic composition analysis

Following taxonomic assignment and taxonomic status identification, the differentially abundant taxon of specific composition within each group was obtained. Different taxonomic levels (phylum, class, order, family, genus and species) were equivalent to community composition at different levels. The analysis methods used include Metastats analysis, LEfSe (linear discriminant analysis effect size) analysis, phylogenetic tree and hierarchical tree construction.

#### Linear discriminant analysis effect size analyses

To compare and visualize significant differences in microbiota between dMMR and pMMR groups, LEfSe analyses were performed with the software LEfSe to identify potential differentially microbial biomarkers using the default parameters. Significance levels for LEfSe were *p* < 0.05 and LDA > 2.0.

#### Correlation network analysis

In order to assess the taxonomic relatedness/association within the gut microbiota, correlation networks were constructed using the SparCC algorithm Python package to represent both co-abundance networks and co-exclusion networks between the top 50 abundant species in all samples. The pseudocount value in SparCC was set to 10–6. The correlation coefficient cutoff of 70 was determined using random matrix theory-based methods implemented in R package RMThreshold. The correlation values with value of *p* < 0.05 and correlation values *r* > 0.9 were retained.

#### KEGG pathways analysis

By using PICRUSt v1.0.0 (Phylogenetic investigation of communities by reconstruction of unobserved states), metabolic and signaling pathways were predicated based on KEGG pathway enrichment analysis of 16S rRNA gene data.

### Statistical analysis

The statistical analysis was conducted using IBM SPSS Statistics version 24.0 (SPSS Inc.). Statistical difference between dMMR and pMMR groups were assessed by Student’s t-test for continuous variables and Pearson’s χ2 test for categorical variables. The difference between OTUs and microbial species were calculated by rank sum tests. To analyze the sequences of microbial community, a Kruskall-Wallis or Mann–Whitney test was utilized to compare the OTUs and taxonomy abundances. Kaplan–Meier method was used to analyze the relationship between MMR status and progress free survival (PFS) or overall survival (OS) in the patients with proximal and distal CRC.

## Results

### MMR status and clinicopathological characteristics of the study subjects

**Figure 1:**
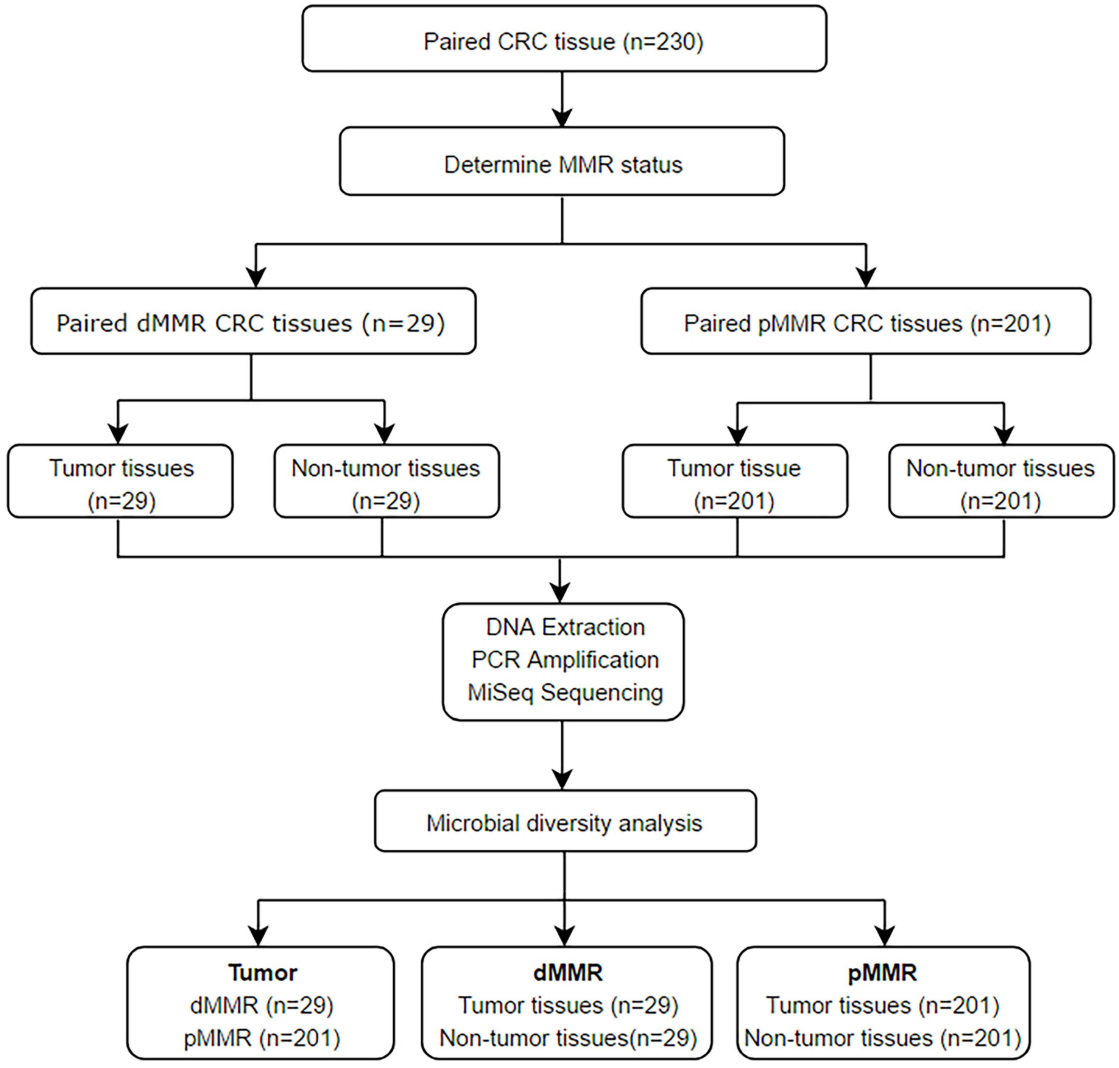
Flowchart of the proposed framework.

**Figure 2:**
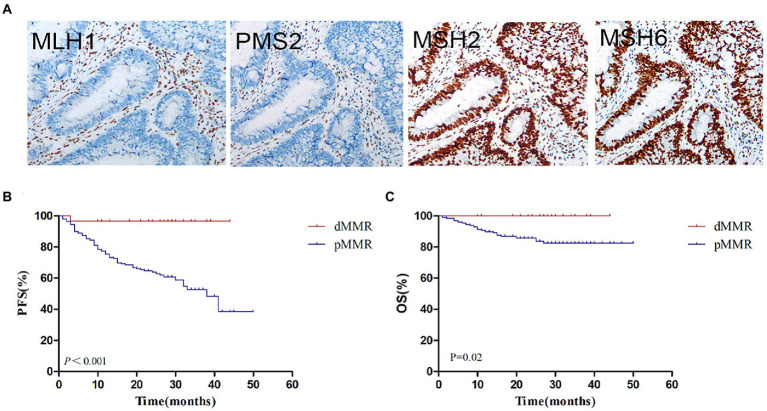
MMR status assessment and its relationship with survival. **(A)** Immunohistochemistry for MMR proteins of MLH1, PMS2, MSH2 and MSH6. **(B)** Kaplan–Meier plot of progression-free survival in CRC patients with dMMR and pMMR status. **(C)** Kaplan–Meier plot of overall survival in CRC patients with dMMR and pMMR status. dMMR, deficient DNA mismatch repair; pMMR, proficient DNA mismatch repair.

The flow chart of this study is shown in Figure 1. According to IHC staining of MMR protein (Figure 2A), 29 cases (12.61%) were classified as dMMR and 201 cases (87.39%) were classified as pMMR (Table 1). In the dMMR group, PMS2, MSH2, and MSH6 were lost at the rate of 0.87, 0.43, and 1.30%, respectively. MSH2 and MSH6 were both lost in 3.04%, while MLH1 and PMS2 were both lost in 6.54% of dMMR subjects. The loss of MSH2, MSH6 and PMS2 without MLH1 was seen in 0.43% of dMMR subjects. The demographic and basic clinical characteristics of the subjects were displayed in Table 2. Between the dMMR and pMMR groups, significant differences were observed in tumor location, tumor size, differentiation, pN stage, American Joint Committee on Cancer (AJCC) TNM stage, distant metastases, tumor nodules and nerve invasion, while there were no statistically significant differences in age, gender, pathological type, pT Stage, liver metastasis, vascular tumor embolus and KRAS/NRAS/BRAF status (*p* > 0.05). Furthermore, compared to pMMR tumors, dMMR status was associated with longer PFS (27.00 vs. 20.00 months; *p* < 0.001) and longer OS (27.00 vs. 23.00; *p* < 0.001) (Figures 2B–C).

**Table 1:** MLH1, MSH2, MSH6, and PMS2 protein expression in colorectal cancers.

	dMMR (*N* = 29)						pMMR (*N* = 201)
MLH1	+	+	+	+	−	+	+
MSH2	+	−	+	−	+	−	+
MSH6	+	+	−	−	+	−	+
PMS2	−	+	+	+	−	−	+
Total *N* (%)	2 (0.87%)	1 (0.43%)	3 (1.30%)	7 (3.04%)	15 (6.52%)	1 (0.43%)	201 (87.39%)

**Table 2:** Clinicopathologic features of the CRC patients with dMMR and pMMR status.

Characteristics	dMMR (N = 29)	pMMR (N = 201)	*p*-value
Age			0.157
<65	25	149	
≥ 65	4	52	
Gender			0.425
Male	19	116	
Female	10	85	
Tumor location			0.002^a^
Proximal	24	100	
Distant	5	101	
Tumor size (cm)			0.001^a^
<4 cm	2	77	
<8 cm，≥4 cm	21	108	
≥ 8 cm	6	16	
Pathological type			0.134
Bulging	15	73	
Infiltrative	1	29	
Ulcerative	13	99	
Differentiation			0.013^a^
Poor	4	28	
Moderate	14	152	
Well	4	7	
Unknown	7	14	
pT stage			0.443
T1-2	3	13	
T 3–4	26	188	
pN stage			<0.001^a^
N0	22	92	
N1	5	60	
N2	2	49	
AJCC TNM stage			<0.001^a^
I-II	22	78	
III-IV	7	123	
Liver metastasis			0.061
No	29	179	
Yes	0	22	
Distant metastases			0.030^a^
No	28	161	
Yes	1	40	
Tumor nodules			0.035^a^
No	27	147	
Yes	2	54	
Nerve invasion			<0.001^a^
No	27	109	
Yes	2	92	
Vascular tumor embolus			
No	23	141	
Yes	6	60	
KRAS status			0.352
Mutant	8	59	
Wild type	5	64	
Unknown	16	78	
NRAS status			0.362
Mutant	0	8	
Wild type	12	115	
Unknown	17	78	
BRAF status			0.898
Mutant	1	9	
Wild type	11	114	
Unknown	17	78	

AJCC, American Joint Committee on Cancer; dMMR, deficient mismatch repair; pMMR, proficient mismatch repair; KRAS, kirsten rat sarcoma viral oncogene; NRAS, neuroblastoma rat sarcoma viral oncogene.

a Data show statistically significant values.

### Gut microbiome diversity analysis

**Figure 3:**
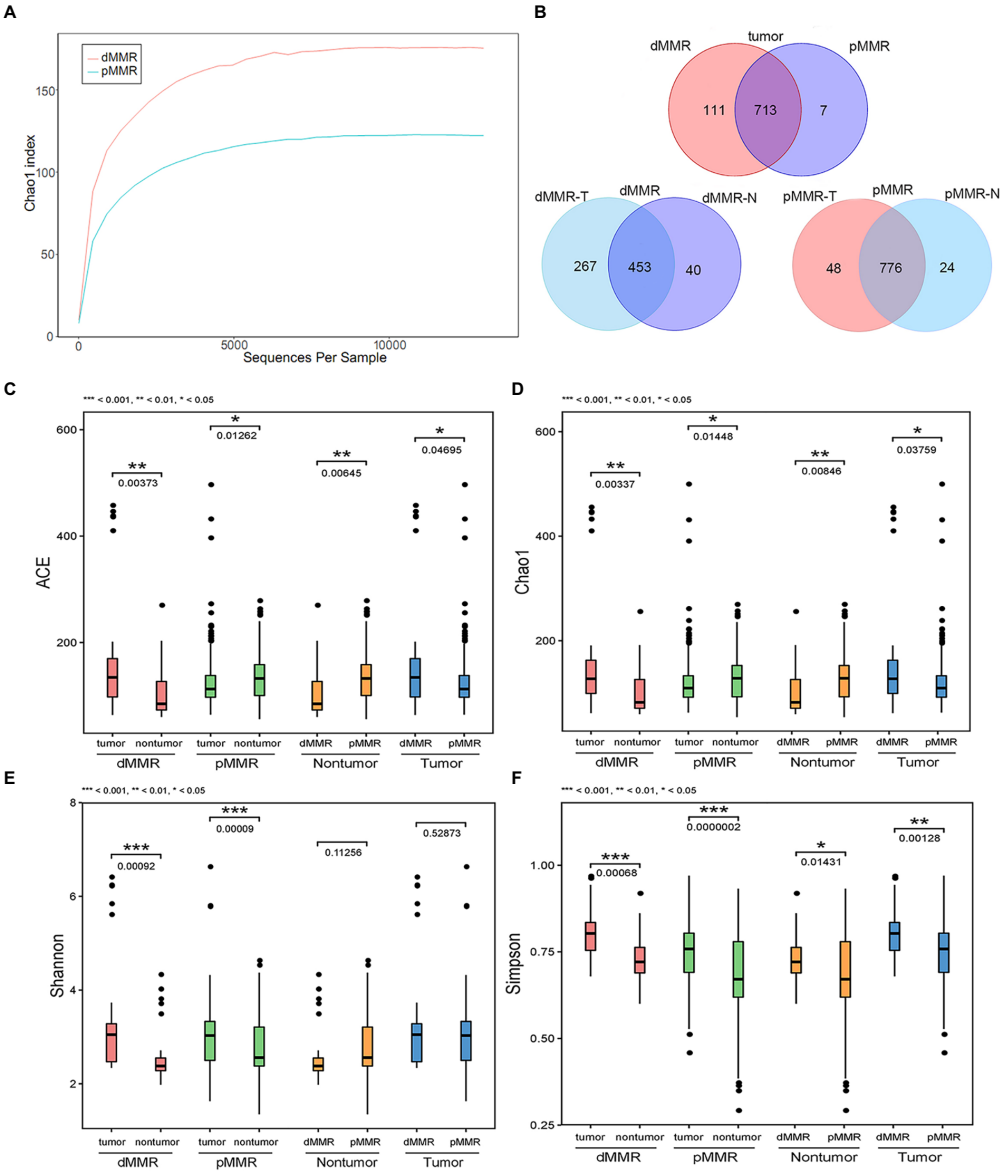
Comparative analysis of alpha diversity between dMMR and pMMR groups. The analysis was performed on the 16S rRNA gene sequencing results. **(A)** Rarefaction curve analysis in the tumor tissue microbiota between dMMR and pMMR. **(B)** Venn diagram showing the shared OTUs in all plots. ACE diversity plot **(C)**, Chao1 diversity plot **(D)**, Shannon diversity plot **(E)**, and Simpson diversity plot **(F)** among the dMMR and pMMR groups. dMMR, deficient DNA mismatch repair; pMMR, proficient DNA mismatch repair; dMMR-T, dMMR tumor tissue samples; pMMR-T, pMMR tumor tissue samples; dMMR-N, dMMR normal paracancerous samples; pMMR-N, pMMR normal paracancerous samples. ***<0.001; **<0.01; *<0.05.

To investigate the gut microbiome diversity between pMMR and dMMR status in CRC patients, we used 16S rRNA gene sequencing to analyze the tumor and normal para-cancerous tissue samples between dMMR and pMMR groups. The sequencing produced 35,350 high-quality reads for each sample with an average length of 338 bp. Rarefaction curve analysis showed that the sequencing depth basically approached saturation in all samples (Figure 3A). Moreover, the Venn diagram illustrating the overlapping OTU data across groups showed that approximately, 824 and 720 OTUs were identified in dMMR and pMMR CRC, respectively. seven of the total 831 OTUs were unique for pMMR tumor tissues and 111 of the total 831 OTUs were unique for dMMR tumor tissues (Figure 3B).

The ACE estimator, Chao1 estimator, Shannon, and Simpson indices of alpha-diversity were used to evaluate the within-sample diversity and richness of the microbiomes in dMMR group and pMMR group (Figures 3C–F and Table 3). The four indices analysis revealed that in dMMR CRC, the were greater diversity and richness in dMMR-T than in normal dMMR-N. In pMMR CRC, the richness of pMMR-T was lower than that of pMMR-N based on ACE and Chao1 estimators, but the diversity of pMMR-T was higher based on Shannon and Simpson indices.

**Table 3:** Microbial alpha-diversity index comparisons.

Index	dMMR-T	dMMR-N	pMMR-T	pMMR-N	*P*-value (dMMR-T vs. pMMR-T)	*P*-value (dMMR-T vs. dMMR-N)	*P*-value (pMMR-T vs. pMMR-N)
Chao1	173.733 ± 127.235	117.709 ± 79.705	122.138 ± 54.750	126.265 ± 44.211	0.038^a^	0.003^a^	0.014‑
Simpson	0.804 ± 0.083	0.748 ± 0.077	0.742 ± 0.088	0.673 ± 0.134	0.001^a^	<0.001^a^	<0.001^a^
Shannon	3.417 ± 1.289	2.812 ± 0.838	2.994 ± 0.642	2.751 ± 0.658	0.529	<0.001^a^	<0.001^a^
ACE	177.992 ± 125.865	121.683 ± 79.592	126.999 ± 55.779	131.137 ± 44.211	0.047^a^	0.004^a^	0.013^a^

dMMR-T, tumor tissues with deficient mismatch repair; dMMR-N, paracancerous tissues with deficient mismatch repair; pMMR-T, tumor tissues with proficient mismatch repair; pMMR-N, paracancerous tissues with proficient mismatch repair.

a Data show statistically significant values.

Notably, the richness and diversity of the microbiome were significantly higher in dMMR-T than in the pMMR-T group according to the comparisons of the ACE estimator, Chao1 estimator and Simpson index. The Shannon index was slightly elevated in the dMMR-T without statistical significance. In addition, the richness of microbiome was lower in dMMR-N than in pMMR-N group. These findings suggested that gut microbial diversity strongly correlated with MMR status.

### Intestinal microbiome composition analysis

To determine the beta-diversity of intestinal microbiota composition, we applied PCoA by using unweighted and weighted UniFrac distances to represent scale of difference among the groups. Table 4 indicated significant dissimilarity in microbiota composition among the groups using the UniFrac unweighted distance method. The tumor tissues and normal paracancerous tissues in both dMMR and pMMR (dMMR-T vs. dMMR-N; pMMR-T vs. pMMR-N) also displayed significant dissimilarity in microbiota composition using weighted distance method. PCoA plots displayed the definite separation of the clusters between among the groups (Supplementary Figure S1).

**Table 4:** Microbial beta-diversity index comparisons.

	Group comparison	*F*	*R* ^2^	*P*-value
Unweighted UniFrac	dMMR-T vs. pMMR-T	8.635	0.03649	0.001^a^
	dMMR-T vs. dMMR-N	2.3293	0.05448	0.007^a^
	pMMR-T vs. pMMR-N	16.581	0.0398	0.001^a^
Weighted UniFrac	dMMR-T vs. pMMR-T	8.4912	0.0359	0.001^a^
	dMMR-T vs. dMMR-N	1.6242	0.03997	0.080
	pMMR-T vs. pMMR-N	20.798	0.04942	0.001^a^

dMMR-T, tumor tissues with deficient mismatch repair; dMMR-N, paracancerous tissues with deficient mismatch repair; pMMR-T, tumor tissues with proficient mismatch repair; pMMR-N, paracancerous tissues with proficient mismatch repair.

a Data show statistically significant values.

**Figure 4:**
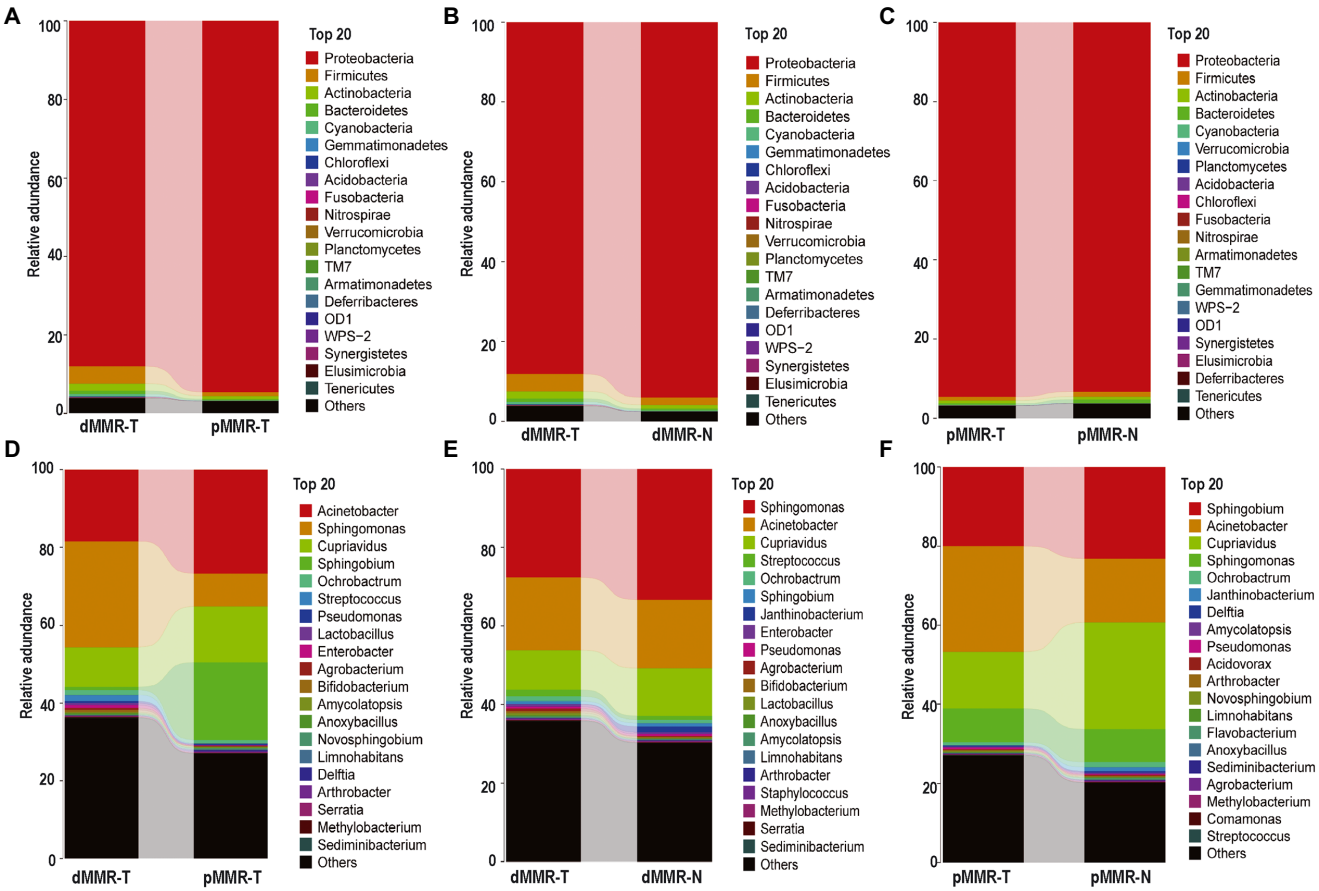
Taxonomic characteristics of the microbiota among the groups. Structure and abundance of flora at the phylum level between dMMR-T and pMMR-T group **(A)**, between dMMR-T and dMMR-N group **(B)**, and between pMMR-T and pMMR-N group **(C)**. dMMR, deficient DNA mismatch repair; pMMR, proficient DNA mismatch repair; dMMR-T, dMMR tumor tissue samples; pMMR-T, pMMR tumor tissue samples; dMMR-N, dMMR normal paracancerous samples; pMMR-N, pMMR normal paracancerous samples. And structure and abundance of flora at the genus level between dMMR-T and pMMR-T group **(D)**, between dMMRT and dMMR-N group **(E)**, and between pMMR-T and pMMR-N group **(F)**.

According to OTU classification results, specific species abundance at phylum and genus level were analyzed. Most of the species in the tissue samples between different groups belonged to Proteobacteria, Firmicutes, Actinobacteria, and Bacteroidetes, accounting for more than 95.1% of the total intestinal flora (Figure 4C). Other bacteria with less abundance, such as Fusobacteria and Verrucomicrobia, were also present in tissue samples. There were differences in the proportion of main intestinal flora between dMMR-T and pMMR-T (Figure 4A), with Proteobacteria accounting for 88.031 and 94.614%, Firmicutes accounting for 4.382 and 0.905%, and Actinobacteria accounting for 1.820 and 0.787%, respectively. Additionally, differences in the relative abundance of Fusobacteria (0.044% vs. 0.009%) and Verrucomicrophyla (0.038% vs. 0.004%) were observed between the dMMR-T and pMMR-T groups. Among dMMR-T and dMMR-N, Proteobacteria accounted for 88.086 and 93.981%, Firmicutes accounted for 4.3785 and 1.8424%, and Actinobacteria accounted for 1.812 and 1.004%, respectively (Figure 4B). The other bacteria species such as Cyanobacteria, Fusobacteria and Verrucomicrobia accounted for 0.475, 0.0442, and 0.038% in dMMR-T, respectively, while accounted for 0.190, 0.006, and 0.003% in dMMR-N, respectively. Among pMMR-T and pMMR-N (Figure 4C), Proteobacteria accounted for 88.031 and 94.614%, Firmicutes accounted for 4.382 and 0.905%, Actinobacteria accounted for 0.787 and 0.758%, respectively. Other phyla of Cyanobacteria, Fusobacteria and Verrucomicrobia accounted for 0.1437, 0.009, and 0.005% in pMMR-T, respectively, while accounted for 0.161, 0.006, and 0.021% in dMMR-T, respectively. Multiple alignment also revealed considerable difference between different samples at the genus level (Figures 4D–F). The microbial community structure according to the classification hierarchy is shown in Supplementary Figure S2.

### Phylum-level changes of the microbial communities

**Figure 5:**
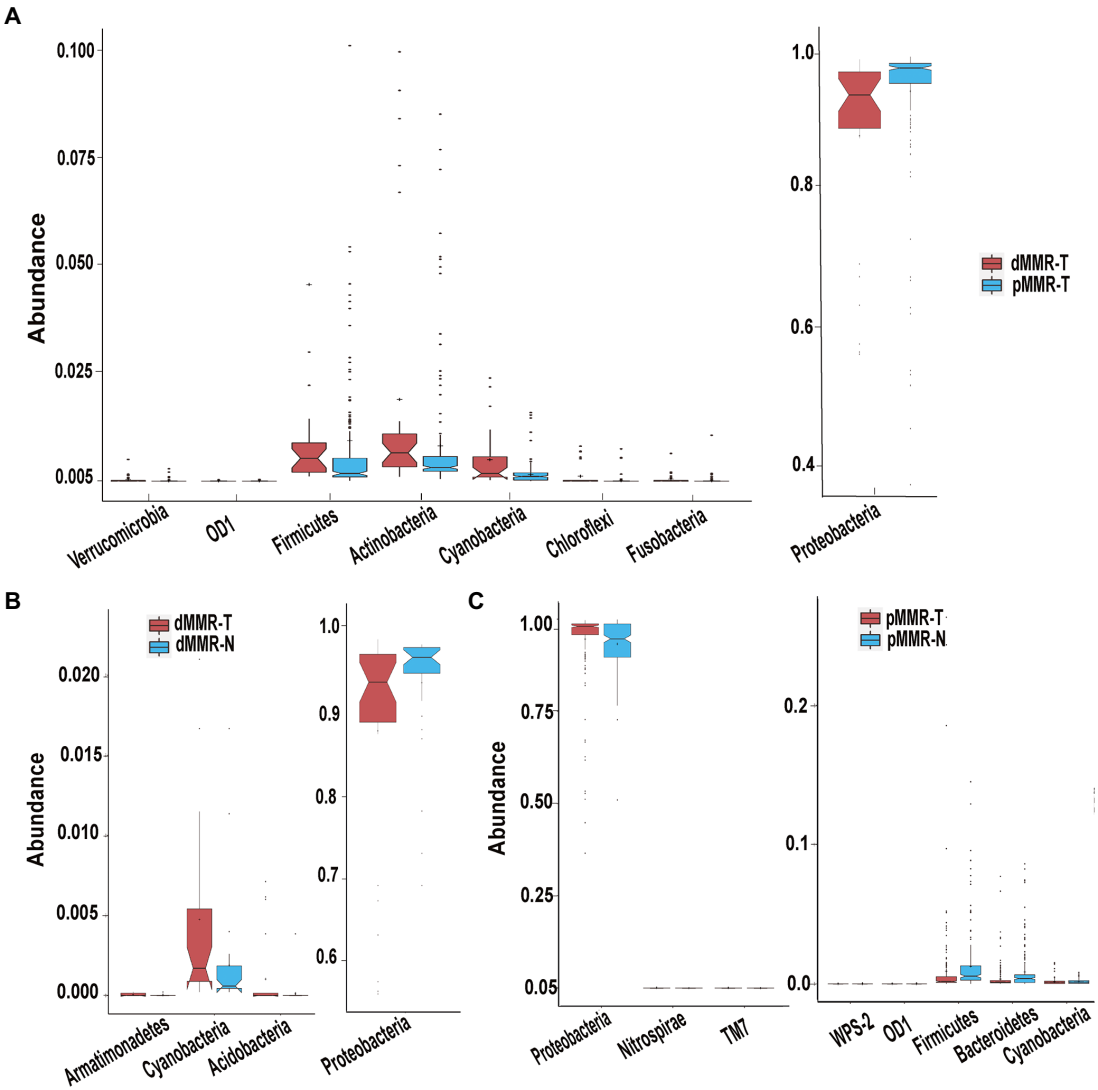
Different phyla among the dMMR and pMMR groups. Increased Phyla (**A**, left panel) and decreased Phyla (**A**, right panel) in dMMR-T group compared to pMMR-T group. Increased Phyla (**B**, left panel) and decreased Phyla (**B**, right panel) in dMMR-T group compared to dMMR-N group. Increased Phyla (**C**, left panel) and decreased Phyla (**C**, right panel) in pMMR-T group compared to pMMR-N group.

The significant changes at the phylum-level among the groups were further analyzed, showing the following results: (i) Firmicutes, Actinobacteria, Fusobacteria and Verrucomicrobia were significantly enriched in dMMR-T, but Proteobacteria abundance was reduced when compared to pMMR-T (Figure 5A). (ii) Compared with dMMR-N, dMMR-T were significantly enriched for Cyanobacteria, Armatimonadetes and Acidobacteria, while Proteobacteria was significantly decreased (Figure 5B). (iii) Compared with pMMR-N, pMMR-T were significantly enriched for Proteobacteria and Nitrospirae, while Firmicutes, Bacteroidetes and Cyanobacteria was significantly diminished (Figure 5C). Moreover, since an elevated Firmicutes/Bacteroidetes ratio has been proven to be a biomarker for gut bacterial dysbiosis (Magne et al., 2020), we found that the Firmicutes/Bacteroidetes ratio in dMMR-T group far exceeded that in the pMMR-T group (*p* < 0.001). The increasing trend of Firmicutes/Bacteroidetes ratio was also observed in dMMR-T compared with dMMR-N, but the difference was not statistically significant (*p* > 0.05) (Supplementary Figure S3).

### Genus-level change of the microbial communities

**Figure 6:**
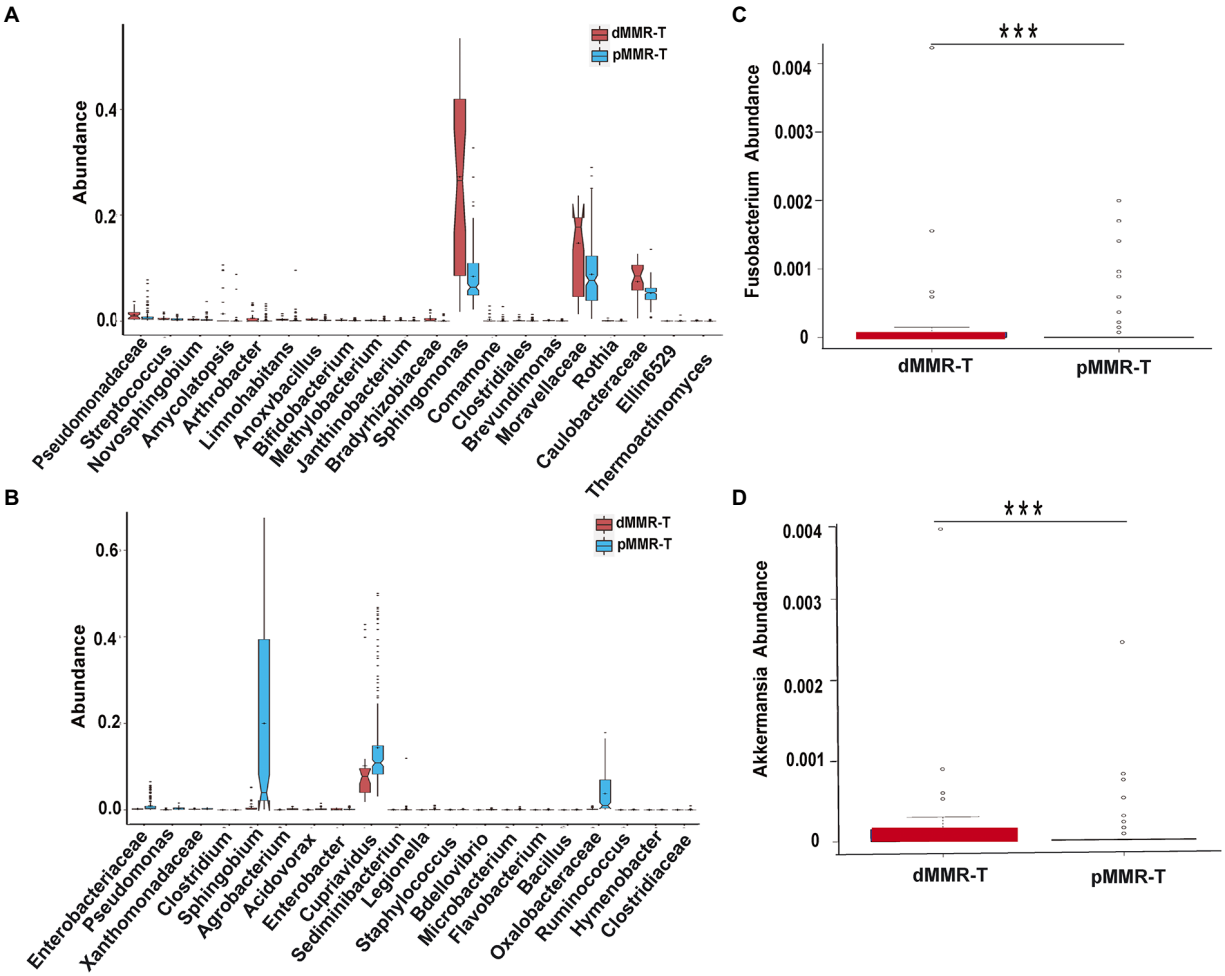
Different genera among the dMMR and pMMR groups. Increased genera **(A)** in dMMR-T group compared to pMMR-T group. Decreased genera **(B)** in dMMR-T group compared to pMMR-T group. The abundance of Fusobacterium was significantly different between dMMR-T and pMMR-T group **(C)**. The abundance of Akkermansia was significantly different between dMMR-T and pMMR-T group **(D)**. *** < 0.001.

We next investigated the genus-level variation between dMMR-T and pMMR-T, and then performed the cluster heatmap analysis. The results revealed: (i) Ochrobactrum, Streptococcus, Pseudomonas, Lactobacillus, Anoxybacillus, Bifidobacterium, Methylobacterium, Staphylococcus, Enterococcus, Rothia, Propionibacterium, Faecalibacterium, Prevotella, Veillonella, Akkermansia, Lactococcus, Ruminococcus, and Coprococcus were more abundant in dMMR-T than in the pMMR-T group, (Figure 6A and Supplementary Figure S4). Notably, the opposite effect was observed for Sphingobium, Cupriavidus and Serratia. (Figure 6B and Supplementary Figure S4). Curiously, the relative abundance of Fusobacterium and Akkermansia in dMMR-T was significantly higher than that of pMMR patients, with significant statistical significance (*p* < 0.001, Figures 6C,D). (ii) Compared with dMMR-N, Sutterella, Blautia, Prevotella, Rothia, Methylobacterium, Akkermansia, Lactobacillus, and Ruminococcus were enriched in dMMR-T (Supplementary Figures S5, S6). (iii) Compared with pMMR-N, Bifidobacterium, Pseudomonas，Microbacterium, Enterobacter were increased in pMMR-T (*P*<0.001), while Clostridium, Coprococcus, Lactobacillus, Blautia, Lactococcus, Roseburia, Oscillospira, Prevotella, Helicobacter, Ruminococcus and Serratia were decreased in pMMR-T (Supplementary Figures S7, S8).

**Figure 7:**
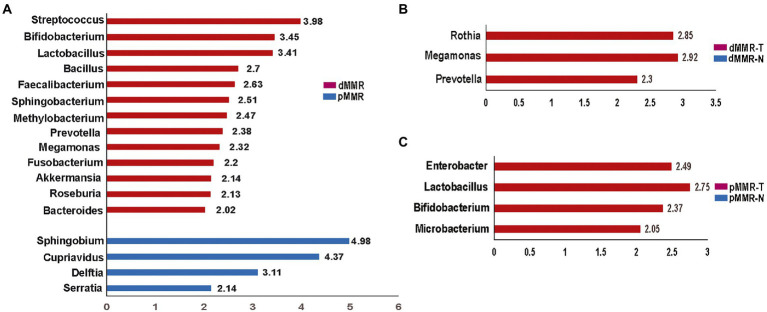
Histogram of the LDA scores computed for features by linear discriminant analysis (LDA) effect size (LEfSe) analysis showing bacteria that were altered among the groups. Key taxa with an LDA of >2 are represented between dMMR-T and pMMR-T group **(A)**, between dMMR-T and dMMR-N group **(B)**, and between pMMR-T and pMMR-N group **(C)**. dMMR-T, dMMR tumor tissue samples (red); dMMR, deficient DNA mismatch repair; pMMR, proficient DNA mismatch repair; pMMR-T, pMMR tumor tissue samples (blue); dMMR-T, dMMR normal paracancerous samples (red); pMMR-T, pMMR normal paracancerous samples (blue).

### The linear discriminant analysis

To identify credible predominant differential flora distinguishing between dMMR and pMMR, we applied the Linear Discriminant Analysis (LDA) score using Lefse algorithm (Figure 7). Flora with an average abundance of less than 0.01% were excluded. In comparison to pMMR-T, dMMR-T group had more predominant taxonomic communities, including Fusobacterium (LDA = 2.20), Akkermansia (LDA = 2.14), Bifidobacterium (LDA = 3.45), Faecalibacterium (LDA = 2.63), Streptococcus (LDA = 3.98) Prevotella (LDA = 2.38), Megamonas (LDA = 2.32), Roseburia (LDA = 2.13), Bacteroides (LDA = 2.02), Bacillus (LDA = 2.70), Lactobacillus (LDA = 3.41). Sphingobacterium (LDA = 2.51) and Methylobacterium (LDA = 2.47). While Serratia (LDA = 2.14), Delftia (LDA = 3.11), Cupriavidus (LDA = 4.37) and Sphingobium (LDA = 4.98) were predominant in pMMR-T group (Figure 7A and Supplementary Figure S9).

Compared to dMMR-N, the dMMR-T group was predominantly enriched in Prevotella (LDA = 2.30, *p* = 0.01), Megamonas (LDA = 2.92) and Rothia (LDA = 2.85) (Figure 7B and Supplementary Figure S10). Compared to pMMR-N, the pMMR-T group was predominantly enriched in Microbacterium (LDA = 2.05), Bifidobacterium (LDA = 2.37), Lactobacillus (LDA = 2.75) and Enterobacter (LDA = 2.49) (Figure 7C and Supplementary Figure S11). These results indicated that CRC patients with dMMR or pMMR exhibited significantly different phylogenetic types of gut microbiotas, which may help to clinically distinguish MMR status.

### Correlation network analysis of related dominant species

**Figure 8:**
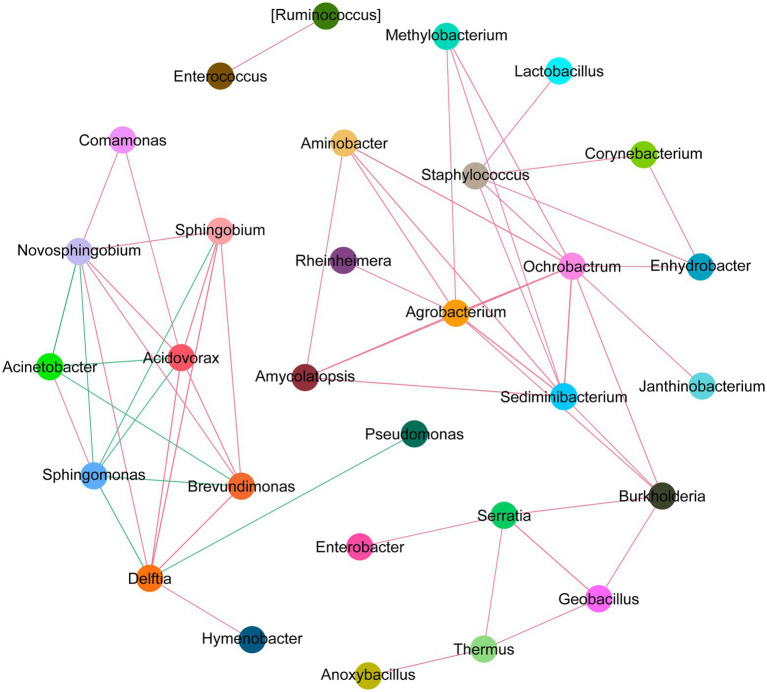
Network analysis diagram of species interaction in CRC with different MMR status. The circles in different colors indicate the dominant bacteria genera, and the lines between the circles indicate that the correlation between the two bacteria genera is significant (*p* < 0.05). The red line represents positive correlation, and the green line represents negative correlation.

To perform an in-depth assessment of the potential interactions among different microbial community members, we constructed the *correlation* network among dominant microbial groups. Based on the SparCC algorithm, we investigated the correlation among top 50 abundant species in different MMR status. We observed that intestinal microflora formed a cross-linked network (Figure 8). Delftia has a strong competitive relationship with Pseudomonas and Sphingomonas, but has a symbiotic relationship with Sphingobium. Enterococcus and Ruminococcus had a strong positive correlation, and Enterobacter and Serratia had strong synergy. This reflects the potential roles these microbes can play as symbionts in the MMR pathway.

### KEGG pathways analysis

**Figure 9:**
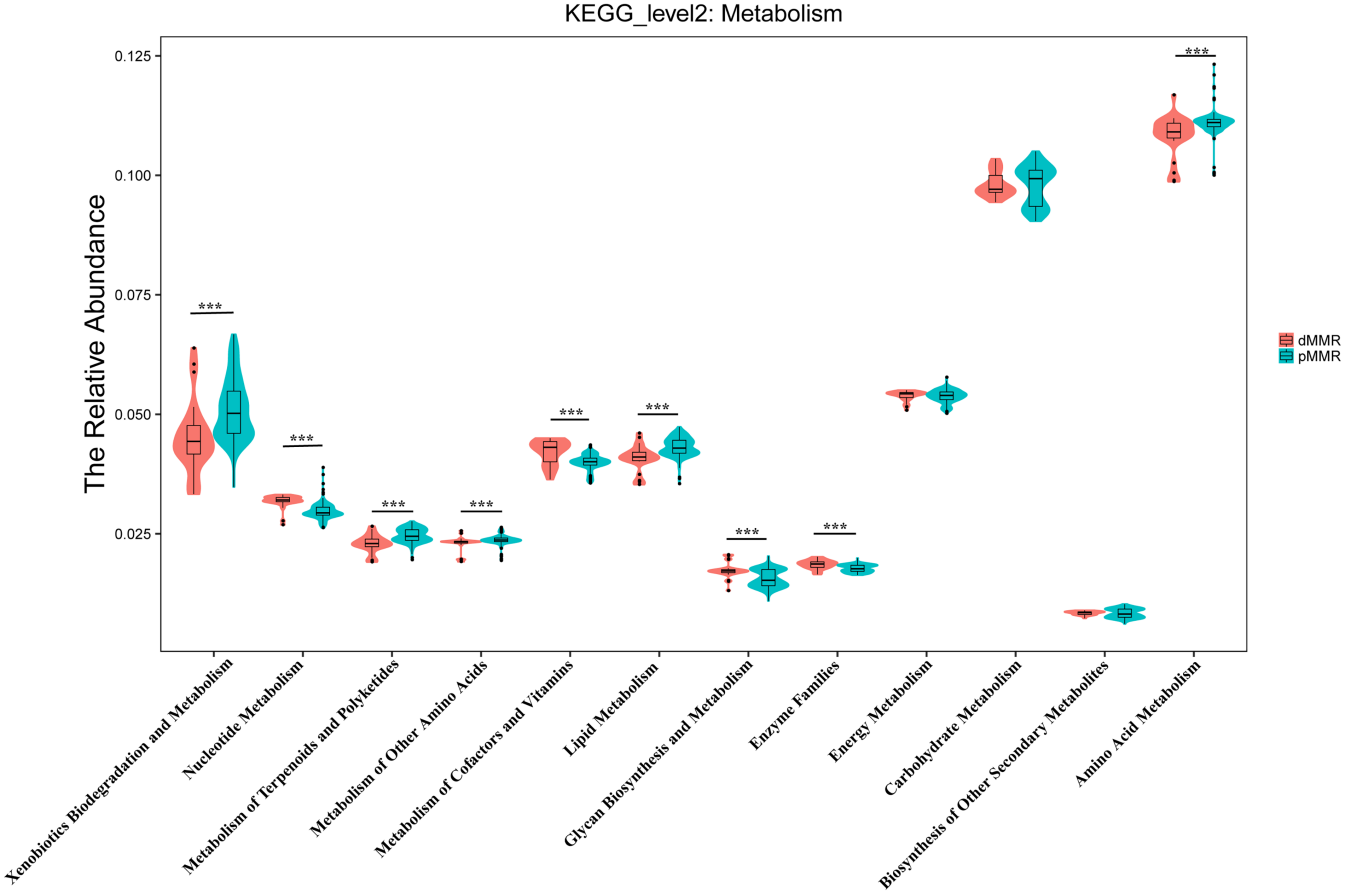
Predictive analysis of secondary metabolic functional pathways of intestinal flora between dMMR and pMMR patients. dMMR, dMMR tumor tissue samples (red); pMMR, pMMR tumor tissue samples (green). The abscissa represents the secondary functional groups of KEGG pathway, and the ordinate represents the relative abundance of samples. *** < 0.001. dMMR, deficient DNA mismatch repair; pMMR, proficient DNA mismatch repair.

Furthermore, we used PICRUST analysis to predict the metabolic function of intestinal flora based on 16S rRNA sequencing results from dMMR and pMMR patients. To explore the possible functional pathways involved, we mapped KO categories to the KEGG pathways. We found 120 KEGG pathways with statistical differences between the dMMR and pMMR groups (Supplementary Table S1). At the level of KEGG secondary pathway, the microbiological function in dMMR group was associated with the biosynthesis and metabolic pathways of glycan, vitamins and nucleotide, cell growth and death pathways, and genetic information replication and repair pathways. The microbiological function in pMMR group was associated with lipid metabolic pathway, terpenoid and polyketone metabolic pathway, amino acid metabolic pathway and membrane transport pathway (*p* < 0.01) (Figure 9). The above results reflect that CRC patients with different MMR status exhibited different metabolic function of intestinal flora, which may further affect the physiological function of the body. This may indicate potential mechanisms related to intestinal microbes that are involved in the occurrence and development of MMR-related CRC.

## Discussion

The importance of specific microbiota in CRC pathogenesis is becoming more widely recognized, but our understanding of how it relates to MMR status remains limited. Here, we conducted 16S rRNA gene sequencing on paired CRC tissue and normal-adjacent tissue from 230 patients (29 dMMR and 201 pMMR). First, we found that the alpha-diversity of the microbiome was significantly higher in the dMMR-T group than in the pMMR-T group, and that it was also richer than in the dMMR-N group. Second, there were significant differences in the microbiota compositions (beta-diversity) between the dMMR-T and pMMR-T groups, between dMMR-T and dMMR-N groups, and between pMMR-T and pMMR-N groups. Among them, the difference between dMMR-T and pMMR-T groups was especially noticeable. At the phylum level, Firmicutes, Actinobacteria, Fusobacteria and Verrucobacteria was significantly enriched in the dMMR-T group, while Proteobacteria was enriched in the pMMR-T group. At the genus level, the abundance of *Fusobacterium, Akkermansia, Bifidobacterium, Faecalibacterium, Streptococcus* and *Prevotella* were significantly richer in the dMMR-T group, while *Serratia, Cupriavidus* and *Sphingobium* were significantly more abundant in the pMMR-T group. Third, we found that the ratio of *Firmicutes/Bacteroidetes* in dMMR-T was significantly higher than in the pMMR-T group. Correlation network analysis revealed extensive cross-talk among bacterial communities. Moreover, KEGG pathways analysis demonstrated that the microbiota linked pathways also different between dMMR-T and pMMR-T groups. To the best of our knowledge, this is the largest and most comprehensive study of Chinese CRC patients analyzing the relationship between MMR status and host microbiome. This study integrates tumor biology and microbiology in a novel and powerful approach to understanding CRC in Asians with varying MMR status.

DNA mismatch repair deficiency is the landmark feature of MSI, which has frequently been measured to assess loss of protein products including MLH1, MSH2, MSH6, and PMS2 (Li et al., 2015). dMMR/MSI-H has been recognized as a credible biomarker for forecasting tumor behavior and response to anti-PD-1 immunotherapy. However, dMMR patients accounted for less than 20% of CRC cases, representing only a small proportion of patients who benefit from immunotherapy. In the present study, we found the ratio of CRC patients with dMMR status was 12.61% (29/230), which was lower than the results of the Malaysia and India population (14.8%) (Cheah et al., 2014), but higher than the reports of the Hispanic population from Puerto Rico (10.24%) (De Jesus-Monge et al., 2010), the Mashhad population (10.25%) (Goshayeshi et al., 2017) and the Fujian population in Southeast China (Ye et al., 2015). These findings indicate that the proportion of dMMR in CRC varies by country and race. Among the 29 CRC patients with dMMR, 51.70% had combined MLH1 and PMS2 deletions, 24.10% had combined MSH2 and MSH6 deletions, and 3.40% had combined MSH2, MSH6 and PMS2 deletions. The single deletion rate of MSH2, MSH6, PMS2 protein were 6.80, 10.20, and 6.80%, respectively. Moreover, when compared to pMMR cases, dMMR patients had more common tumor localization in the proximal colon, were more at pTNM I-II stage, had larger tumors, and had a decreasing trend of lymph node metastasis, tumor nodules expansion, neurological invasion and distant metastases. This is basically consistent with the characteristics reported by related literatures (Jakstaite et al., 2015; Goshayeshi et al., 2017; Chen et al., 2019). Generally, at stage II and III CRC, dMMR/MSI-H is indicative of better prognosis than pMMR/MSS (Popat et al., 2005). This is further supported by our study showing that patients with dMMR had improved PFS (*p* < 0 0.05) and prolonged OS (*p* < 0.05).

In dMMR tumors, high numbers of somatic mutations occur frequently because of deficient MMR function, leading to synthesis of altered amino acids (ie., neoantigens) (Le et al., 2017). dMMR CRC are often pathologically characterized as the presence of tumor-infiltrating lymphocytes and a Crohn’s-like lymphocytic reaction (Matsukuma et al., 2012). These features make dMMR tumors susceptible to ICIs (Le et al., 2017). In 12 different tumors, including CRC, objective Response Rate (ORR) were observed in 53% of patients and complete responses (CR) were achieved in 21% of patients (Le et al., 2017). Nonetheless, only dMMR tumors respond well to ICIs, while the majority of pMMR patients do not.

Increasing evidence suggests that diversity and abundance of gut microbiota influence the efficacy and toxicity of immunotherapy (Johnson et al., 2016; Dai et al., 2020). According to Gopalakrishnan et al., bacterial diversity significantly correlates with ICI therapy response and patients survival (Velikova et al., 2021). The low microbiome diversity caused by antibiotic overuse has been recognized as a hidden villain behind immunotherapy failure (McQuade et al., 2019). In this study, we consistently found that there were higher species diversity and richness in the dMMR-T than in the pMMR-T. Moreover, the microbial diversity was also increased in dMMR-T group relative to the dMMR-N group. However, in pMMR CRC, the microbial burden in pMMR-T presented inconsistent alfa-diversity using different analysis of indices. The results indicated a direct link between MMR status and gut microbes. Whether the differences in immunotherapy response among patients with different MMR status are associated with the microbial diversity or burden need further investigation.

Until now, a precise list of microorganisms involved in dMMR CRC has remained elusive. A recent study linked CRC microflora to tumor CMS *via* 16S rRNA analysis (Kang et al., 2021). The study revealed that the relative abundances of *Fusobacteria* and *Bacteroidetes* were enriched in CMS1, while *Firmicutes* and *Proteobacteria* were decreased. Given that CMS1 is characterized by dMMR/MSI and immune activation, these species could be involved with dMMR status. Interestingly, Vanessa et al. analyzed 25 CRC patients with dMMR at Mayo Clinic in United States in 2018 to determine the biological variables (Hale et al., 2018). The results showed that *Bacteroides fragilis* and *sulfidogenic F. nucleatum* were significantly enriched in dMMR CRC, but not in pMMR CRC. Of note, Shuji Ogino et al. performed quantitative PCR assay to measure the amount of tissue *F. nucleatum* DNA in 1,069 CRC patients and discovered that *F. nucleatum* enrichment was associated with MSI-H regardless of CIMP and BRAF mutation status (Mima et al., 2016). Tomomitsu et al. consistently confirmed that *F. nucleatum* in CRC tissue was heavily enriched in MSI cases through quantitative real-time PCR (Tahara et al., 2014). In agreement with previous studies, our findings indicated that Fusobacteria at the phylum level and *F. nucleatum* at the genus level were preferentially abundant in the dMMR-T group compared with the pMMR-T group. LEfSe analysis demonstrated that *F. nucleatum* had has a potential predictive value for dMMR, with an LDA value of 2.2.

Notably, the high proportion of certain species in the dMMR-T group include *Akkermansia*, *Bifidobacterium*, *Prevotella*, *Streptococcus*, *Peptostreptococcus*, and *Faecalibacterium*. Matson et al. (2018) found higher relative abundance of a group of eight species driven by *Bifidobacterium longum* in responding (R) compared with non-responding (NR) patients with metastatic melanoma. Routy et al. (2018) found responsiveness to PD-1 therapy is defined by an increased abundance of *Akkermansia muciniphila*. Importantly, FMT from R in mice with tumors showed better response to PDL-1 therapy. Gopalakrishnan et al. (2018) showed higher relative abundance of Faecalibacterium in R than NR patients. Similarly, our study discovered a higher relative abundance of Bifidobacterium, Akkermansia, and Faecalibacterium in dMMR-T than in pMMR-T, with a respective LDA value of 3.15, 2.14 and 2.63. We also found *Proteobacteria* decreased in dMMR-T, in line with the previous report of reduced *Proteobacteria* in the CMS1 subtype.

Additionally, our analysis also showed different microbiota related metabolic pathways between dMMR and pMMR groups. The enriched pathways in the dMMR-T group included glycan biosynthesis and metabolic pathways, nucleotide metabolic pathways, cell growth and death pathways, and genetic replication and repair pathways. However, the pMMR-T group was remarkably enriched in lipid metabolic pathway, and the terpenoid, polyketone and amino acid metabolic pathway. Most recently, Xu et al. also consistently found that the changes of gut microbiome in MSS/pMMR CRC affect the metabolism pathway of glycerol and phospholipid, which may affect the immunotherapeutic potential in the MSS-type CRC tumor-bearing mice model (Xu et al., 2020). These data imply that the different MMR status may differentially impact gut microbes and related signal pathways, leading to varied outcomes in CRC patients. The potential interrelations and mechanisms could be used as indicators for clinical predictions and interventions.

Our study has several limitations. First, because this was a retrospective study, our conclusions may require further validation from prospective studies. Second, we used only IHC method to detect the expression of four MMR proteins, MLH1, MSH2, MSH6, and PMS2 to determine whether a CRC patient was dMMR or pMMR. Although IHC methods have been confirmed to detect the MMR genes reliably (Mishima et al., 2020), IHC may still produce false positive or false negative results due to the variations in antibodies and staining conditions. Third, to investigate the richness and composition of gut microbiome, we used FFPE tissues, which have lower sensitivity and specificity than fresh or frozen tissues due to poor DNA quality. And microbiome clarified in the FFPE tissues only represents the inner resident microbiome of the CRC tissue. Moreover, our microbiota analysis rely on 16sRNA analysis but lack metagenomic and transcriptomic data, which could provide a more comprehensive picture. Furthermore, our study used clinical-based cancer and paracancerous tissue samples rather than the fecal samples, which cannot fully represent the profiles of intestinal microbiota. Finally, because the low incidence of dMMR status in CRC limited the number of dMMR patients included, the findings in the study need to be replicated in more combined research globally. In future, we anticipate that the implementation of the microbiotome will become a game-changing novel therapy for CRC with pMMR status to convert “cold” tumors into “hot” tumors to bring a more efficient immune response.

## Conclusion

In summary, we revealed a significant association between MMR status and gut microbiome profiles in a cohort of Asian CRC patients. The compositions of the Firmicutes, Actinobacteria, Fusobacteria and Verrucobacteria families, and the Fusobacterium, Akkermansia, Bifidobacterium, and Streptococcus genera, differ significantly between dMMR and pMMR patients. These differences in microbiome profiles may contribute to different immunotherapeutic response and clinical outcome in CRC. The dMMR-associated microbiota participate in vital signaling pathways like genetic replication and repair pathways. Future studies on the intervention of the composition of the microbiome in CRC, especially in pMMR non-responders to immunotherapy, will be critical to improve antitumor efficacy of oncology therapeutics.

## Data availability statement

The datasets presented in this study can be found in online repositories. The names of the repositories and accession numbers can be found at: National Center for Biotechnology Information (NCBI), Sequence Read Archive (SRA), https://www.ncbi.nlm.nih.gov/sra, SRR12019460-SRR12020117 and NCBI BioSample, https://www.ncbi.nlm.nih.gov/biosample/, SAMN30428202-SAMN30428209. The other original contributions presented in the study are included in the article/Supplementary material, further inquiries can be directed to the corresponding authors.

## Ethics statement

The studies involving human participants were reviewed and approved by Ethics Committee of Tongji Medical College of Huazhong University of Science and Technology. The patients/participants provided their written informed consent to participate in this study.

## Author contributions

KC, LL, XN, TZ, JF, and HL: conceptualization. MJ, JW, LS, BZ, and FS: formal analysis. HL: funding acquisition. HL, JF, MJ, JW, XC, XD, and SD: investigation. KC, LL, XN, and HL: methodology. HL, KC, XN, and JF: resources. HL, JF, and TZ: supervision. KC, LL, XN, and TZ: visualization. MJ and JW: writing–original draft. HL and JF: writing–review and editing. All authors contributed to the article and approved the submitted version.

## Funding

This work was supported by the Beijing Xisike Clinical Oncology Research Foundation (Y-tongshu2021/ms-0107), National Key R&D Program of China (No. 2018YFC1313300), National Natural Science Foundation of China (Nos. 81702392 and 81472707), Chinese South Western Oncology Group (No. CSWOG-CCET005), Beijing Bethune Public Welfare Foundation (No. BJ-GYQZHX2021006), and Chen Xiao-Ping Foundation for the Development of Science and Technology of Hubei Province (No. CXPJJH122006-1003).

## Conflict of interest

The authors declare that the research was conducted in the absence of any commercial or financial relationships that could be construed as a potential conflict of interest.

## Publisher’s note

All claims expressed in this article are solely those of the authors and do not necessarily represent those of their affiliated organizations, or those of the publisher, the editors and the reviewers. Any product that may be evaluated in this article, or claim that may be made by its manufacturer, is not guaranteed or endorsed by the publisher.

## Supplementary material

SUPPLEMENTARY TABLE S2 Patients clinical information with sample identification.

SUPPLEMENTARY FIGURE S1 Weighted and unweighted UniFrac distance PCoA plots of changes in microbiome composition between dMMR and pMMR tumors and their noraml paracancerpus tissues. **(A,B)** Unweighted **(A)** and weighted **(B)** UniFrac distance PCoA diagram of dMMR-T and pMMR-T group; **(C,D)** WEIGHTED **(C)** and unweighted **(D)** UniFrac distance PCoA diagram of dMMR-T and dMMR-N group; **(E,F)** UNweighted **(E)** and weighted **(F)** UniFrac distance PCoA diagram of pMMR-T and pMMR-N group. Each dot represents a sample, and dots with different colors belong to different sample groups. The percentage of the three-dimensional coordinate axis represents the percentage of variance in the original data that can be explained by the corresponding principal coordinate. dMMR, deficient DNA mismatch repair; pMMR, proficient DNA mismatch repair; dMMR-T, dMMR tumor tissue samples; pMMR-T, pMMR tumor tissue samples; dMMR-N, dMMR normal paracancerous samples; pMMR-N, pMMR normal paracancerous samples.

SUPPLEMENTARY FIGURE S2 Chords of dMMR and pMMR CRC tumor samples-species abundance association. Circos analysis directly reflecting the composition proportion of dominant species in each sample at the species level and the distribution proportion of dominant species in different samples. dMMR, deficient DNA mismatch repair; pMMR, proficient DNA mismatch repair.

SUPPLEMENTARY FIGURE S3 Comparison of firmicutes/Bacteroidetes ratios between groups. Comparative analysis of firmicutes/Bacteroidetes ratio between dMMR-T and pMMR-T group (A), between dMMR-T and dMMR-N group (left), and between pMMR-T(middle) and pMMR-N group (right). dMMR, deficient DNA mismatch repair; pMMR, proficient DNA mismatch repair; dMMR-T, dMMR tumor tissue samples; pMMR-T, pMMR tumor tissue samples; dMMR-N, dMMR normal paracancerous samples; pMMR-N, pMMR normal paracancerous samples.

SUPPLEMENTARY FIGURE S4 Cluster heat map of relative abundance of different genera in dMMR and pMMR tumor tissues at genus level.The color depth represents the relative abundance of different bacteria. Strong yellow indicates high relative abundance of the genus, while light blue indicates absence of the genus. The abscissa is the number of included samples, and the ordinate is the name of the different bacteria genus.

SUPPLEMENTARY FIGURE S5 Heat map of relative abundance of the genera in dMMR-T and dMMR-N tissues. The color depth represents the relative abundance of different bacteria. The abscissa is the number of included samples, and the ordinate is the name of the different bacteria genus. dMMR, deficient DNA mismatch repair; dMMR-T, dMMR tumor tissue samples; dMMR-N, dMMR normal paracancerous samples.

SUPPLEMENTARY FIGURE S6 Box plot of the top 20 differences in relative abundance of intestinal genera between dMMR-T and dMMR-N group. dMMR, deficient DNA mismatch repair; dMMR-T, dMMR tumor tissue samples; dMMR-N, dMMR normal paracancerous samples.

SUPPLEMENTARY FIGURE S7 Heat map of relative abundance of different genera in pMMR-T and pMMR-N tissues. The color depth represents the relative abundance of different bacteria. The abscissa is the number of included samples, and the ordinate is the name of the different bacteria genus. pMMR, proficient DNA mismatch repair; pMMR-T, dMMR tumor tissue samples; pMMR-N, dMMR normal paracancerous samples.

SUPPLEMENTARY FIGURE S8 Box plot of top 20 differences in relative abundance of intestinal genera between pMMR-T and pMMR-N. pMMR, proficient DNA mismatch repair; pMMR-T, dMMR tumor tissue samples; pMMR-N, dMMR normal paracancerous samples.

SUPPLEMENTARY FIGURE S9 Phylogenetic branching of bacterial enrichment in dMMR and pMMR tumor samples. The brightness of each point is proportional to its effect size. Taxonomic levels from phylum to genus are indicated by large circles in the center (P: phylum; C: class; O: order; F: Family; G: genus). The diameter of each circle indicates the relative abundance of the flora. dMMR, deficient DNA mismatch repair; pMMR, proficient DNA mismatch repair.

SUPPLEMENTARY FIGURE S10 Phylogenetic branching of bacterial enrichment in dMMR-T and dMMR -N. The brightness of each point is proportional to its effect size. Taxonomic levels from phylum to genus are indicated by large circles in the center (P: phylum; C: class; O: order; F: Family; G: genus). The diameter of each circle indicates the relative abundance of the flora. dMMR, deficient DNA mismatch repair; dMMR-T, dMMR tumor tissue samples; dMMR-N, dMMR normal paracancerous samples.

SUPPLEMENTARY FIGURE S11 Phylogenetic branching of bacterial enrichment in pMMR-T and pMMR-N. The brightness of each point is proportional to its effect size. Taxonomic levels from phylum to genus are indicated by large circles in the center (P: phylum; C: class; O: order; F: Family; G: genus). The diameter of each circle indicates the relative abundance of the flora. pMMR, proficient DNA mismatch repair; pMMR-T, dMMR tumor tissue samples; pMMR-N, dMMR normal paracancerous samples.

## Abbreviations

MMR: Mismatch repair
CRC: Colorectal cancer
dMMR: Deficient mismatch repair
pMMR: Proficient mismatch repair
MSI: Microsatellite instability
FFPE: Formalin-fixed paraffin-embedded
NGS: Next generation sequencing
rRNA: Ribosomal RNA
IHC: Immunohistochemistry
RAS: Rat sarcoma viral oncogene homolog
BRAF: B-rapidly accelerated fibrosarcoma
KRAS: Kirsten Rat Sarcoma viral oncogene homolog
NRAS: Neuroblastoma rat sarcoma viral oncogene homolog
OTUs: Operational taxonomic units
PCoA: Principal coordinate analysis
AJCC: American Joint Committee on Cancer
OS: Overall survival
LDA: Linear discriminant analysis
KEGG: Kyoto encyclopedia of genes and genomes
*F*. *nucleatum*: *Fusobacterium nucleatum*
