# Supplementary figures and images for: Gut microbiota distinct between colorectal cancers with deficient and proficient mismatch repair: A study of 230 CRC patients
Source: Front Microbiol. 2022 Oct 13;13:993285. doi: 10.3389/fmicb.2022.993285 (PMC9607965; doi:10.3389/fmicb.2022.993285)

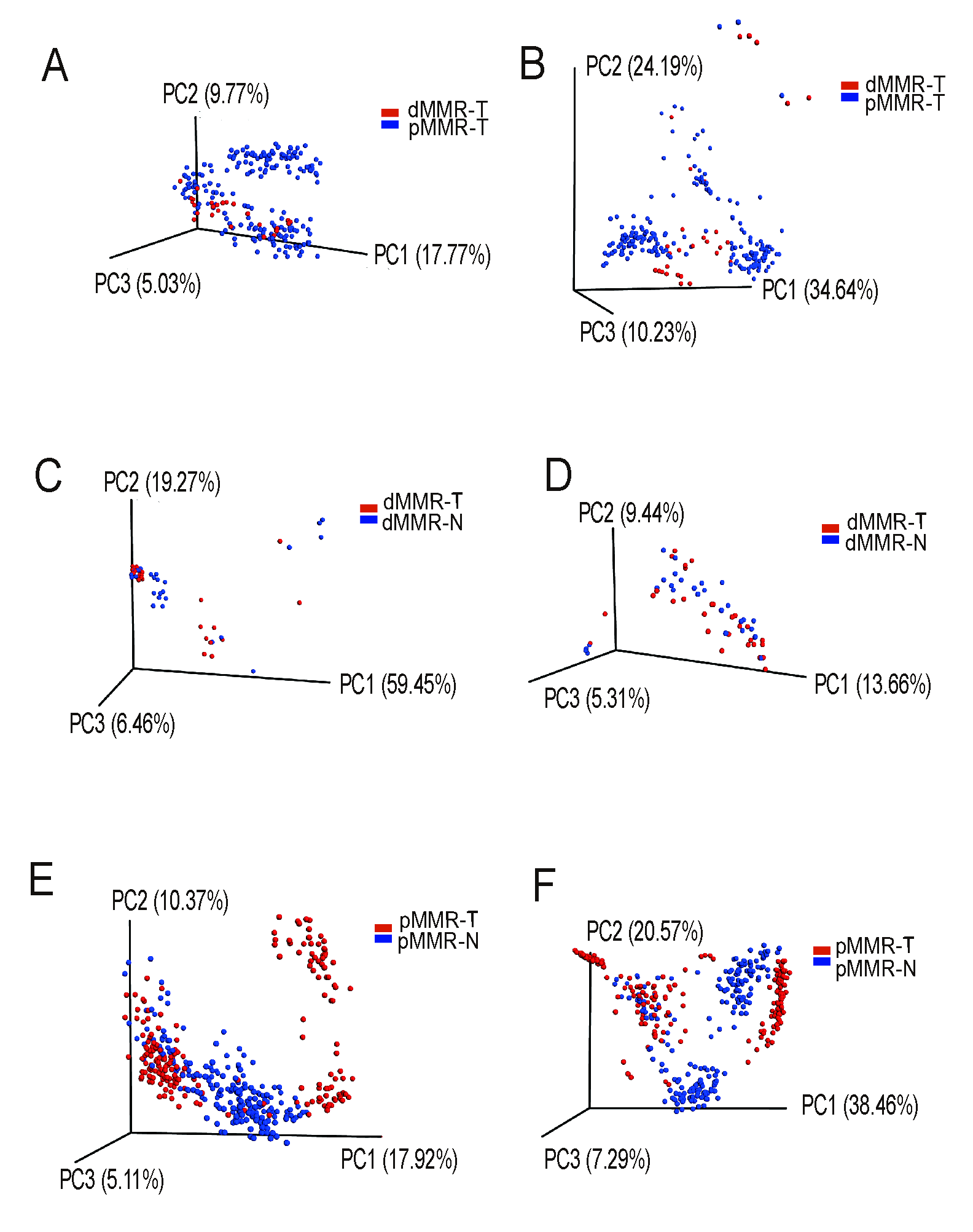

Supplement: SUPPLEMENTARY FIGURE S1 — Weighted and unweighted UniFrac distance PCoA plots of changes in microbiome composition between dMMR and pMMR tumors and their noraml paracancerpus tissues. (A,B) Unweighted (A) and weighted (B) UniFrac distance PCoA diagram of dMMR-T and pMMR-T group; (C,D) WEIGHTED (C) and unweighted (D) UniFrac distance PCoA diagram of dMMR-T and dMMR-N group; (E,F) UNweighted (E) and weighted (F) UniFrac distance PCoA diagram of pMMR-T and pMMR-N group. Each dot represents a sample, and dots with different colors belong to different sample groups. The percentage of the three-dimensional coordinate axis represents the percentage of variance in the original data that can be explained by the corresponding principal coordinate. dMMR, deficient DNA mismatch repair; pMMR, proficient DNA mismatch repair; dMMR-T, dMMR tumor tissue samples; pMMR-T, pMMR tumor tissue samples; dMMR-N, dMMR normal paracancerous samples; pMMR-N, pMMR normal paracancerous samples. [file Image_1.TIF]

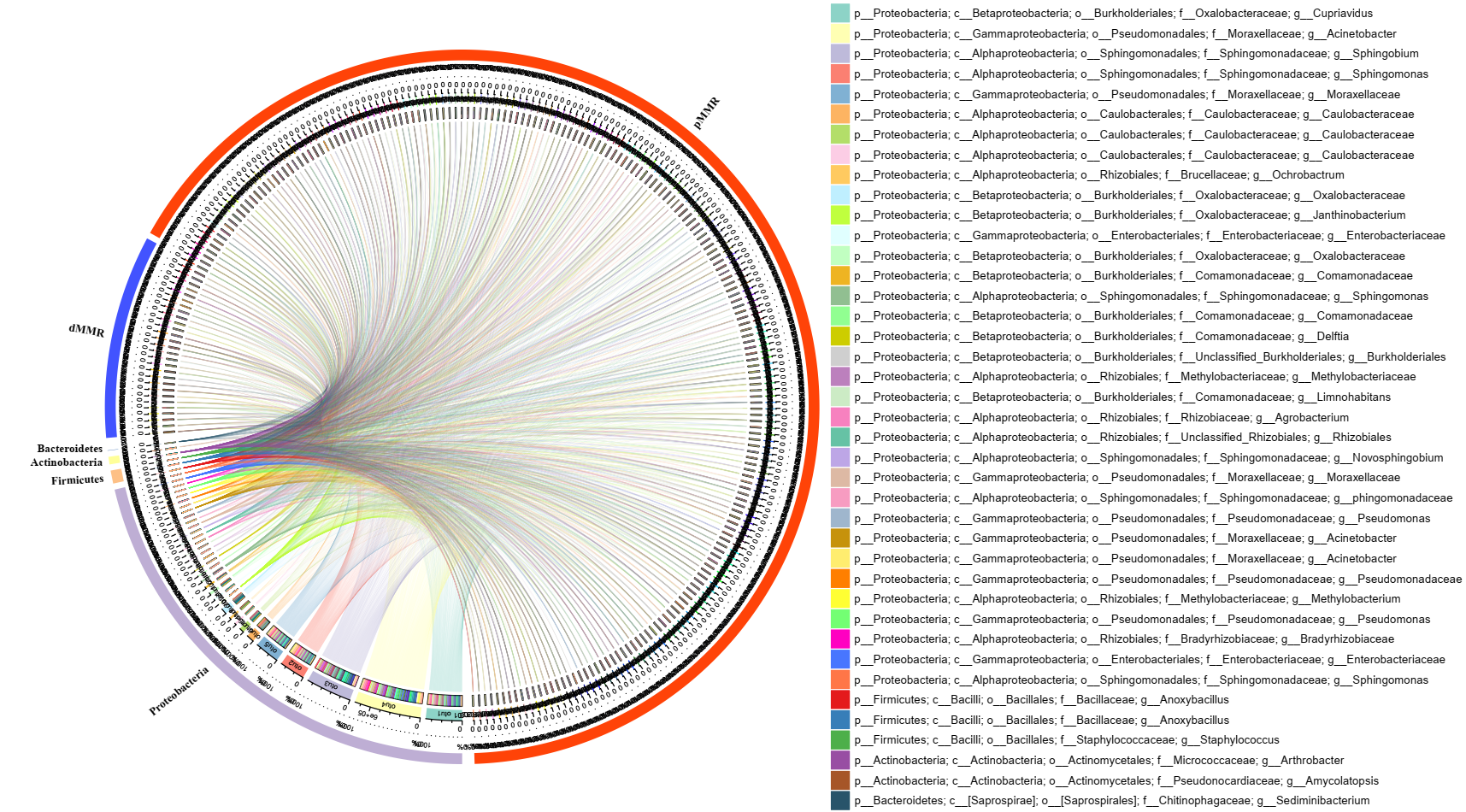

Supplement: SUPPLEMENTARY FIGURE S2 — Chords of dMMR and pMMR CRC tumor samples-species abundance association. Circos analysis directly reflecting the composition proportion of dominant species in each sample at the species level and the distribution proportion of dominant species in different samples. dMMR, deficient DNA mismatch repair; pMMR, proficient DNA mismatch repair. [file Image_2.TIF]

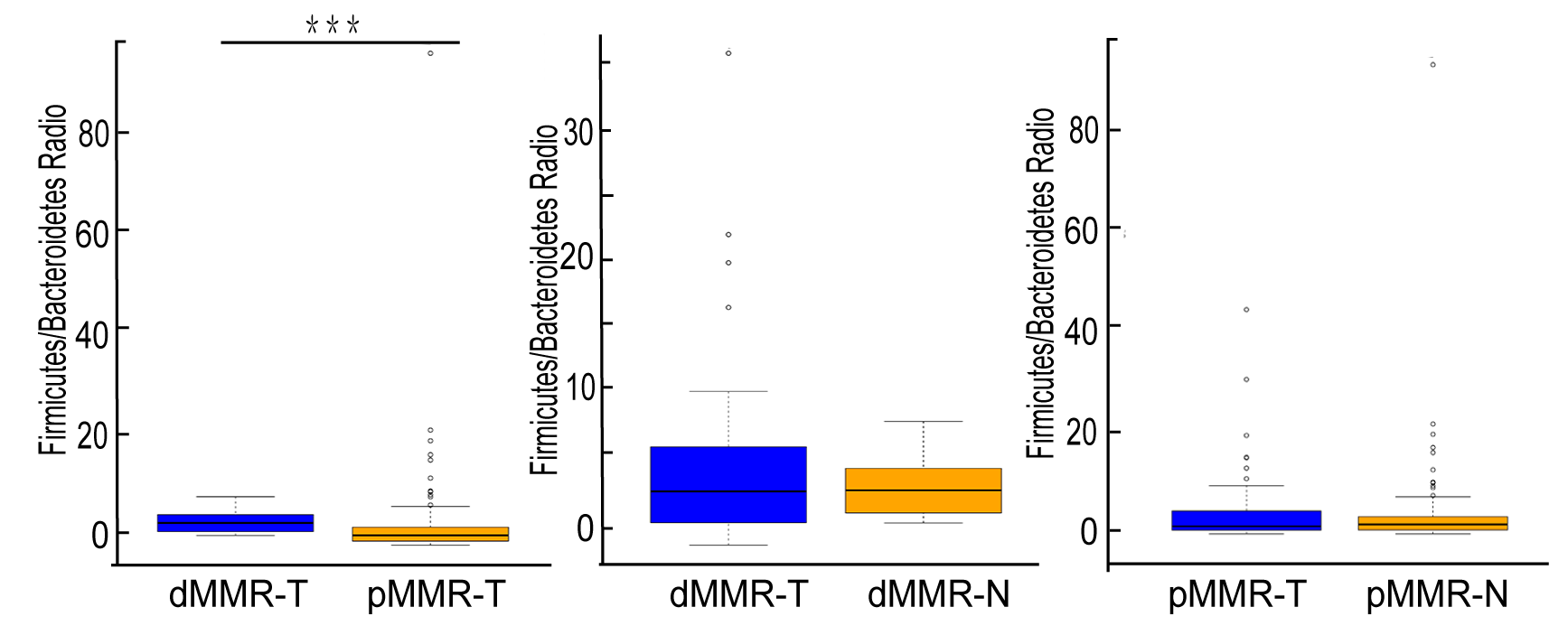

Supplement: SUPPLEMENTARY FIGURE S3 — Comparison of firmicutes/Bacteroidetes ratios between groups. Comparative analysis of firmicutes/Bacteroidetes ratio between dMMR-T and pMMR-T group (A), between dMMR-T and dMMR-N group (left), and between pMMR-T(middle) and pMMR-N group (right). dMMR, deficient DNA mismatch repair; pMMR, proficient DNA mismatch repair; dMMR-T, dMMR tumor tissue samples; pMMR-T, pMMR tumor tissue samples; dMMR-N, dMMR normal paracancerous samples; pMMR-N, pMMR normal paracancerous samples. [file Image_3.TIF]

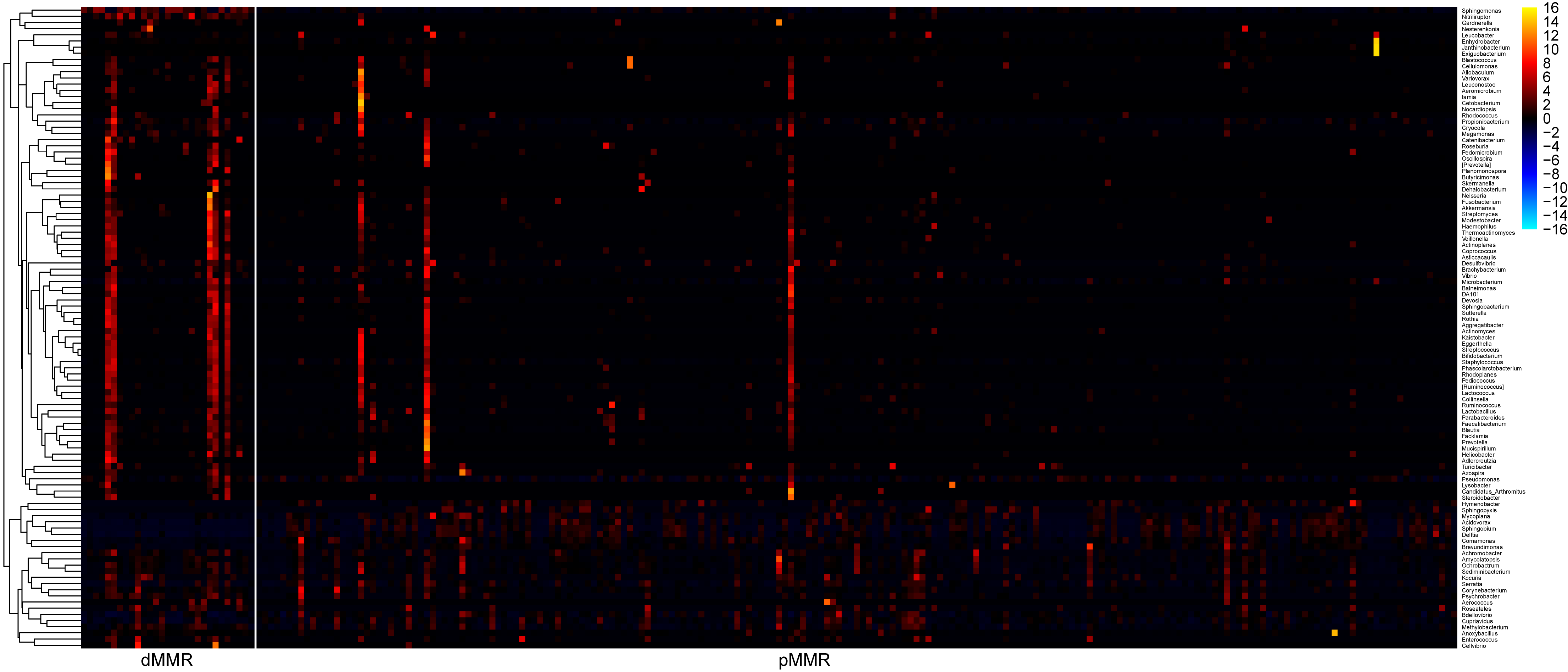

Supplement: SUPPLEMENTARY FIGURE S4 — Cluster heat map of relative abundance of different genera in dMMR and pMMR tumor tissues at genus level.The color depth represents the relative abundance of different bacteria. Strong yellow indicates high relative abundance of the genus, while light blue indicates absence of the genus. The abscissa is the number of included samples, and the ordinate is the name of the different bacteria genus. [file Image_4.TIF]

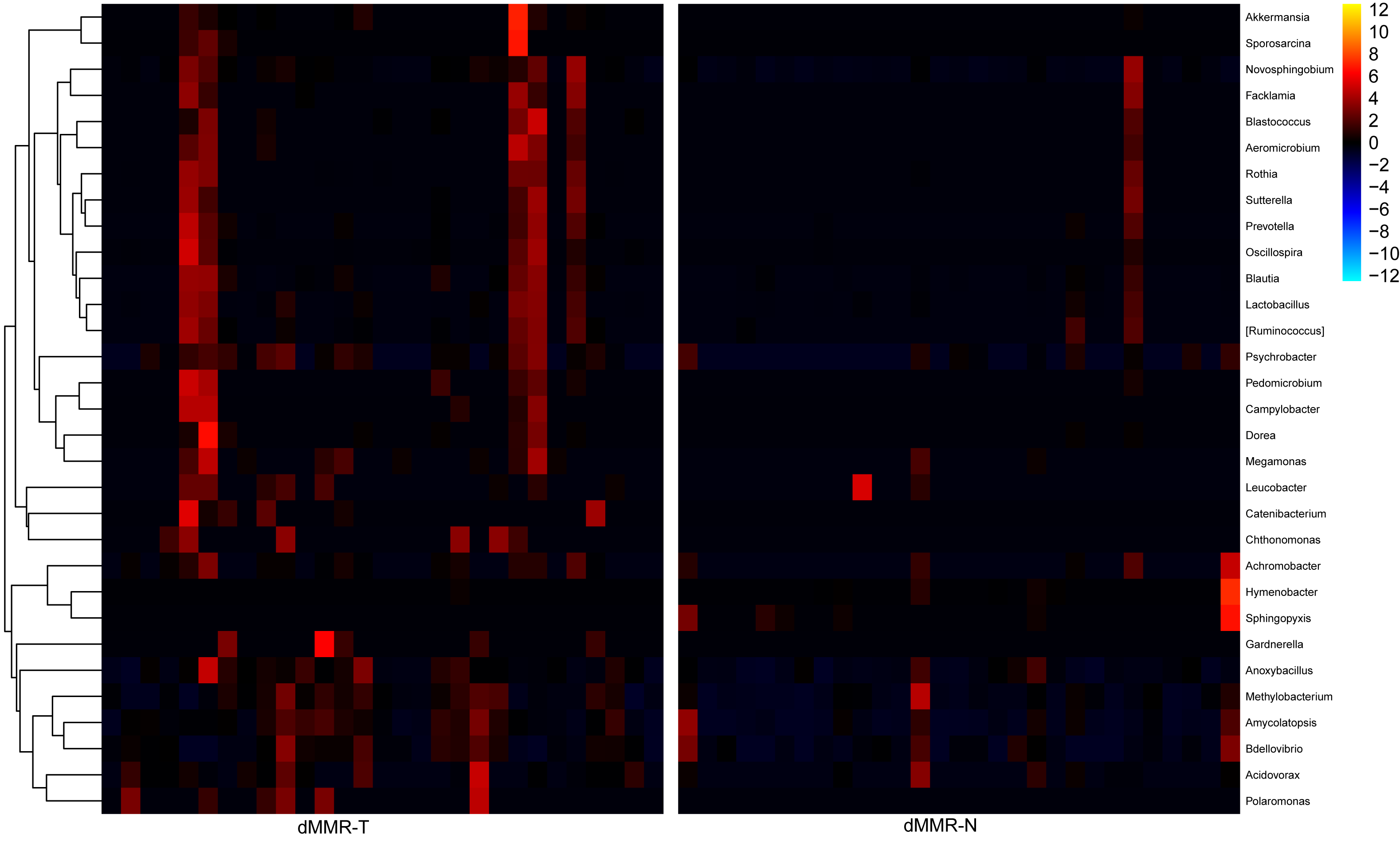

Supplement: SUPPLEMENTARY FIGURE S5 — Heat map of relative abundance of the genera in dMMR-T and dMMR-N tissues. The color depth represents the relative abundance of different bacteria. The abscissa is the number of included samples, and the ordinate is the name of the different bacteria genus. dMMR, deficient DNA mismatch repair; dMMR-T, dMMR tumor tissue samples; dMMR-N, dMMR normal paracancerous samples. [file Image_5.TIF]

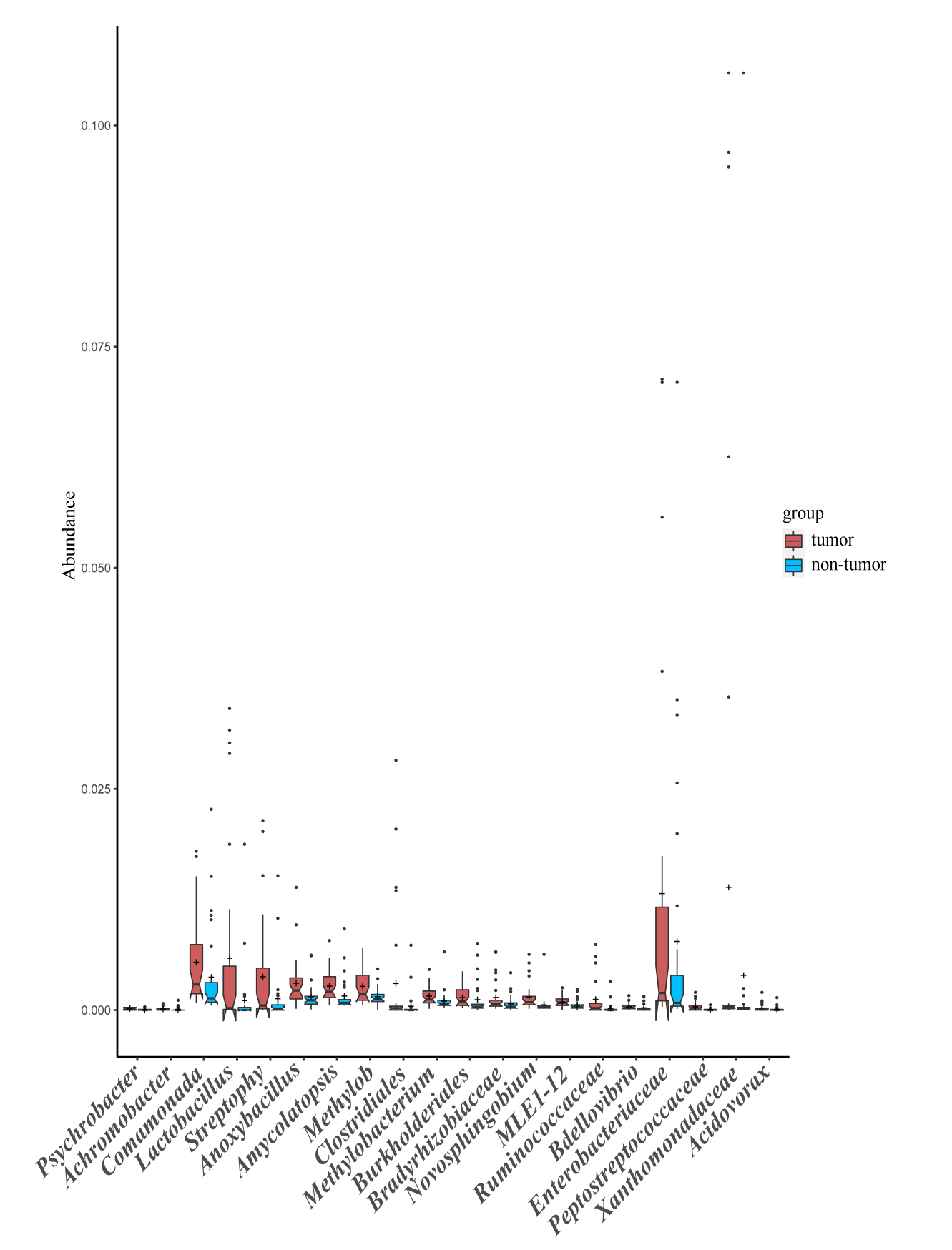

Supplement: SUPPLEMENTARY FIGURE S6 — Box plot of the top 20 differences in relative abundance of intestinal genera between dMMR-T and dMMR-N group. dMMR, deficient DNA mismatch repair; dMMR-T, dMMR tumor tissue samples; dMMR-N, dMMR normal paracancerous samples. [file Image_6.TIF]

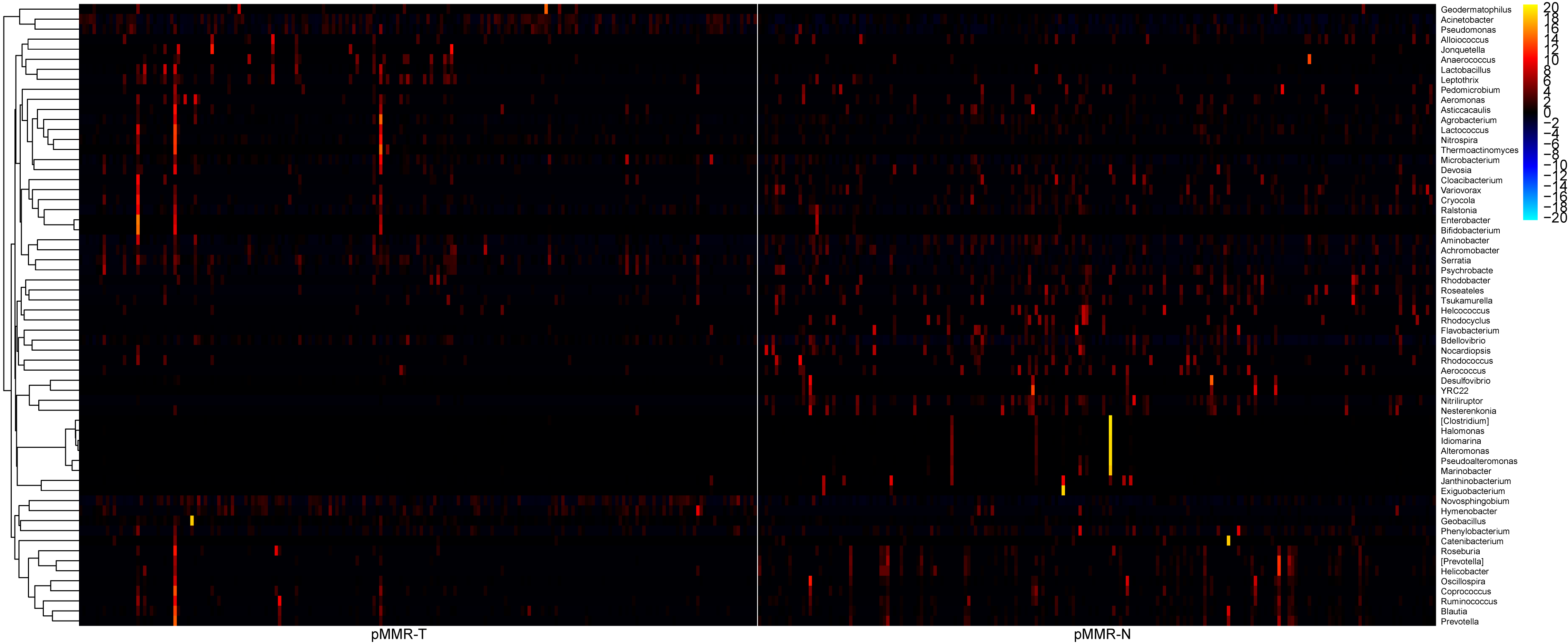

Supplement: SUPPLEMENTARY FIGURE S7 — Heat map of relative abundance of different genera in pMMR-T and pMMR-N tissues. The color depth represents the relative abundance of different bacteria. The abscissa is the number of included samples, and the ordinate is the name of the different bacteria genus. pMMR, proficient DNA mismatch repair; pMMR-T, dMMR tumor tissue samples; pMMR-N, dMMR normal paracancerous samples. [file Image_7.TIF]

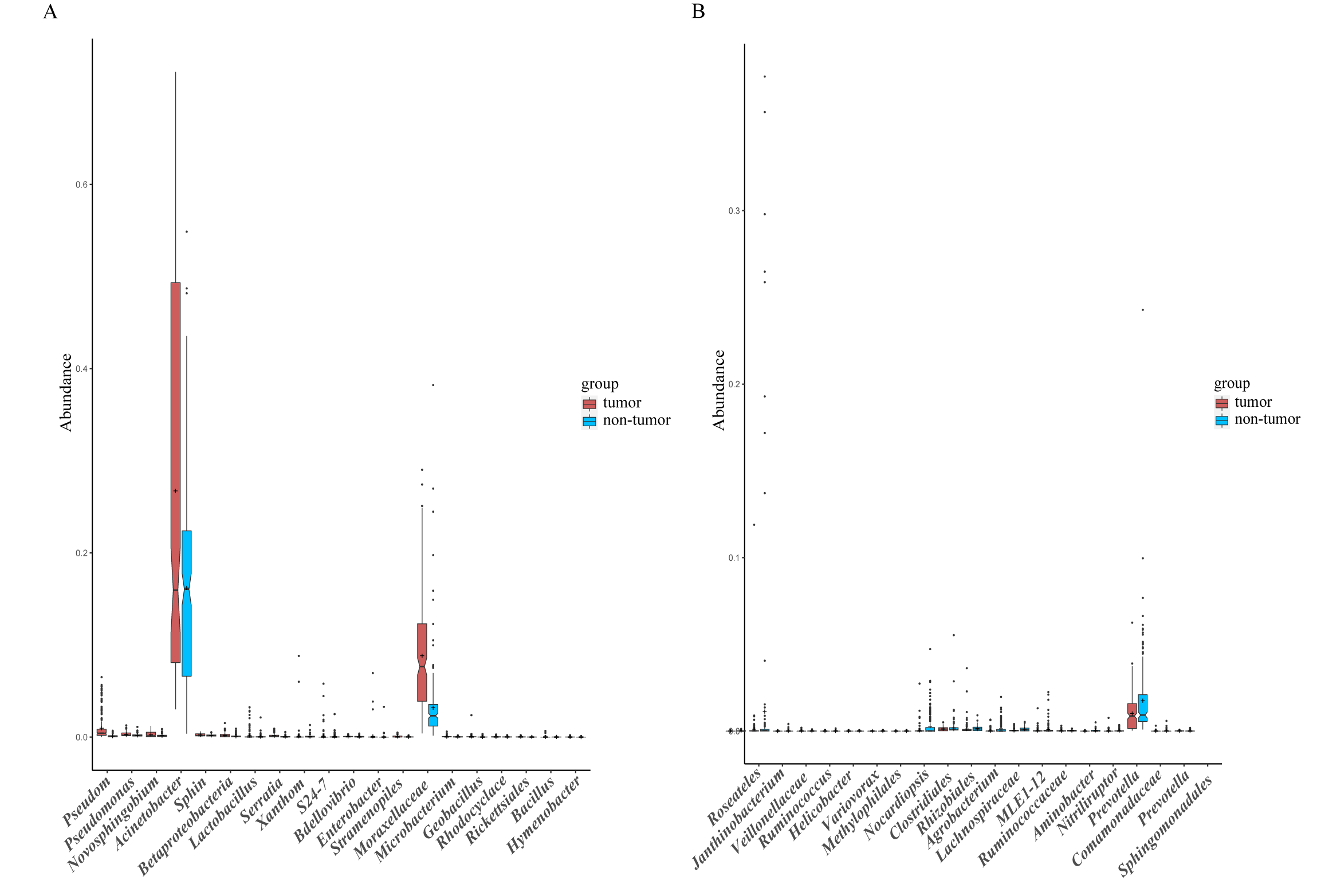

Supplement: SUPPLEMENTARY FIGURE S8 — Box plot of top 20 differences in relative abundance of intestinal genera between pMMR-T and pMMR-N. pMMR, proficient DNA mismatch repair; pMMR-T, dMMR tumor tissue samples; pMMR-N, dMMR normal paracancerous samples. [file Image_8.TIF]

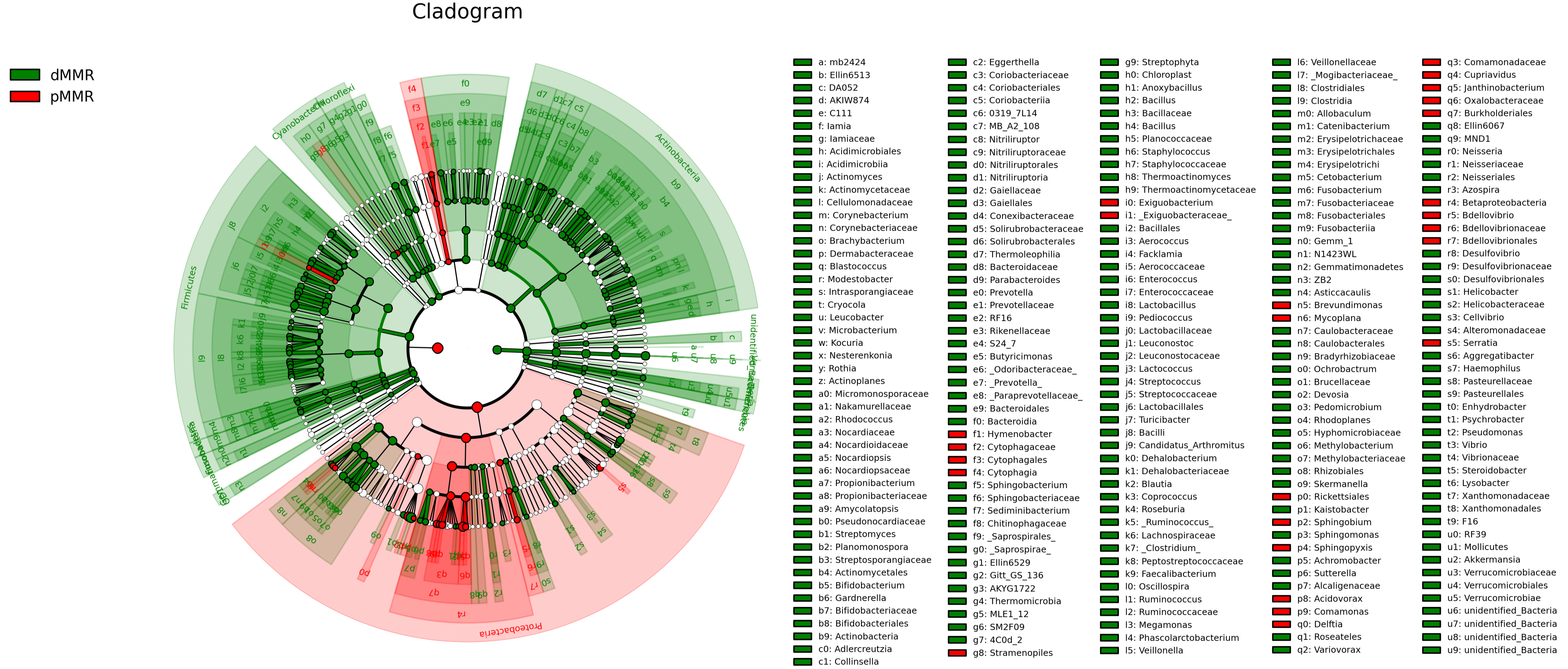

Supplement: SUPPLEMENTARY FIGURE S9 — Phylogenetic branching of bacterial enrichment in dMMR and pMMR tumor samples. The brightness of each point is proportional to its effect size. Taxonomic levels from phylum to genus are indicated by large circles in the center (P: phylum; C: class; O: order; F: Family; G: genus). The diameter of each circle indicates the relative abundance of the flora. dMMR, deficient DNA mismatch repair; pMMR, proficient DNA mismatch repair. [file Image_9.TIF]

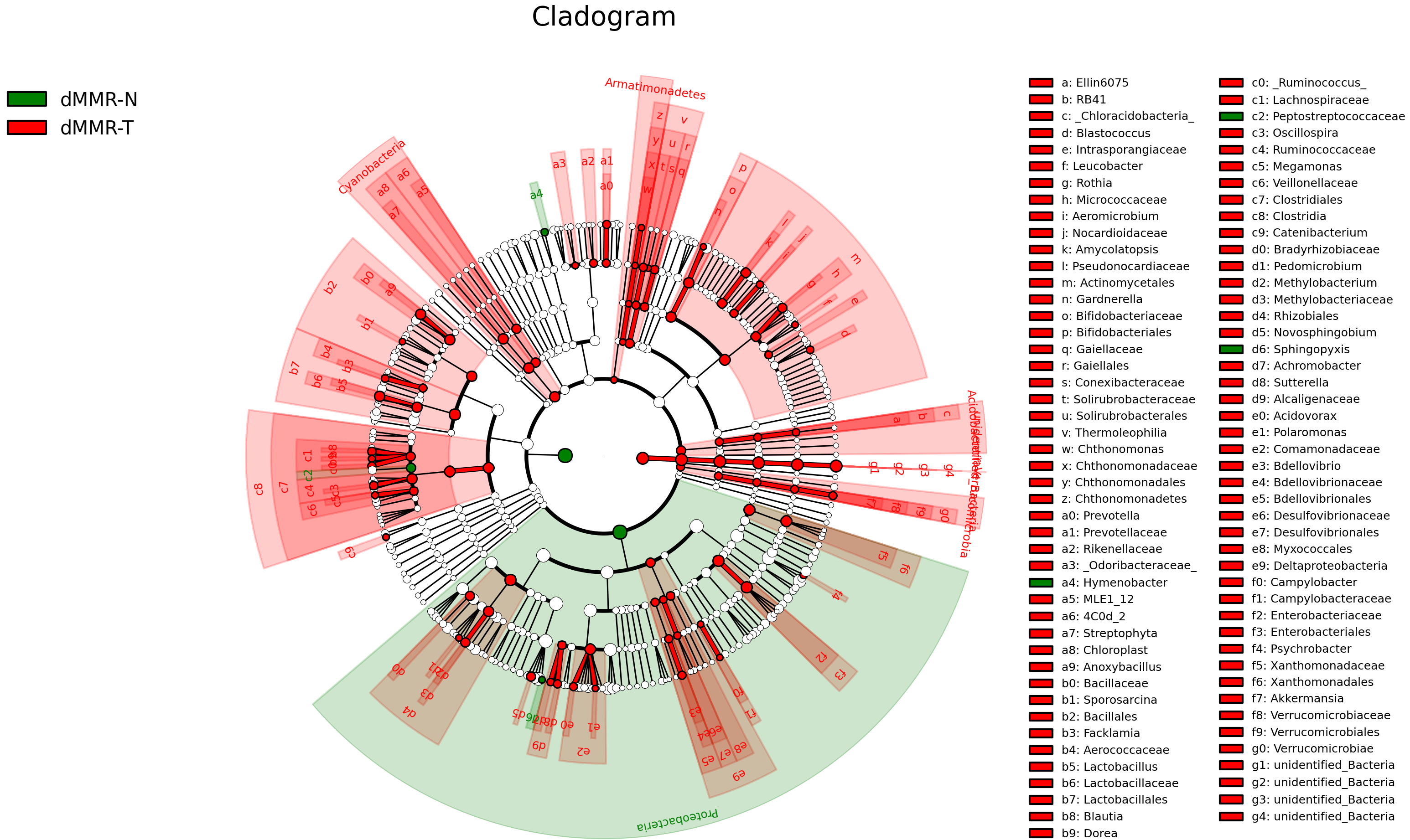

Supplement: SUPPLEMENTARY FIGURE S10 — Phylogenetic branching of bacterial enrichment in dMMR-T and dMMR -N. The brightness of each point is proportional to its effect size. Taxonomic levels from phylum to genus are indicated by large circles in the center (P: phylum; C: class; O: order; F: Family; G: genus). The diameter of each circle indicates the relative abundance of the flora. dMMR, deficient DNA mismatch repair; dMMR-T, dMMR tumor tissue samples; dMMR-N, dMMR normal paracancerous samples. [file Image_10.TIF]

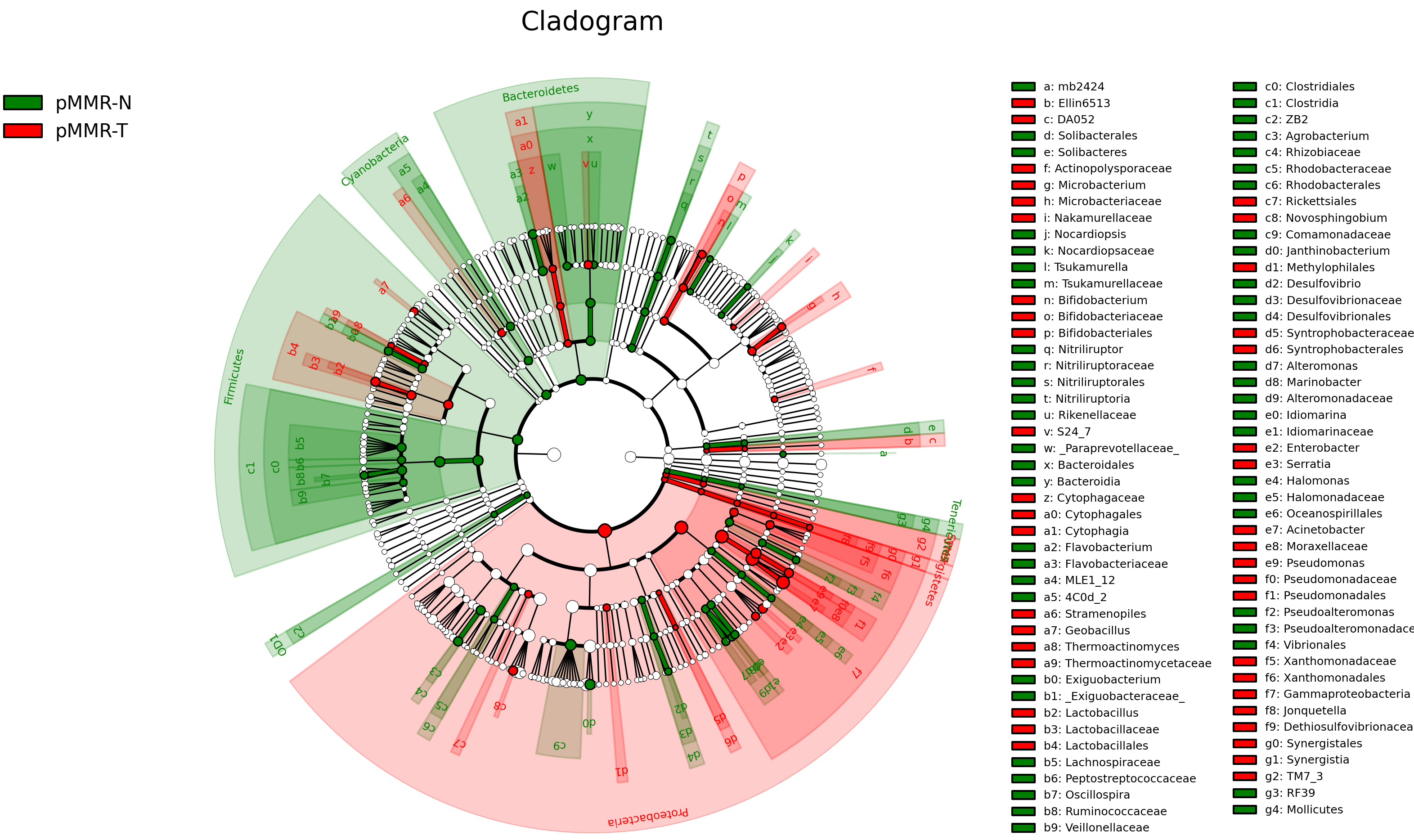

Supplement: SUPPLEMENTARY FIGURE S11 — Phylogenetic branching of bacterial enrichment in pMMR-T and pMMR-N. The brightness of each point is proportional to its effect size. Taxonomic levels from phylum to genus are indicated by large circles in the center (P: phylum; C: class; O: order; F: Family; G: genus). The diameter of each circle indicates the relative abundance of the flora. pMMR, proficient DNA mismatch repair; pMMR-T, dMMR tumor tissue samples; pMMR-N, dMMR normal paracancerous samples. [file Image_11.TIF]
