# Supplementary material for: Gut microbiota distinct between colorectal cancers with deficient and proficient mismatch repair: A study of 230 CRC patients
Source: Front Microbiol. 2022 Oct 13;13:993285. doi: 10.3389/fmicb.2022.993285 (PMC9607965; doi:10.3389/fmicb.2022.993285)
Supplement: Supplementary file 13 [file Table_1.DOC]

**Table S1** KEGG pathways analysis in intestinal microecology between dMMR and pMMR groups

|  | **KEGG description Level 2** | **KEGG description Level 3** | **enriched group** | ***p*-value** |
| --- | --- | --- | --- | --- |
|  | **Transport and Catabolism** |  |  |  |
|  |  | Lysosome | dMMR | ＜0.001 |
|  |  | Peroxisome | pMMR | 0.002 |
|  |  | Phagosome | dMMR | 0.01 |
|  | **Cell Motility** |  |  |  |
|  |  | Bacterial chemotaxis | dMMR | 0.02 |
|  |  | Cytoskeleton proteins | pMMR | 0.01 |
|  |  | Regulation of actin cytoskeleton | dMMR | 0.02 |
|  | **Folding, Sorting and Degradation** |  |  |  |
|  |  | Proteasome | pMMR | 0.02 |
|  |  | Ubiquitin system | dMMR | 0.02 |
|  |  | Basal transcription factors | pMMR | ＜0.001 |
|  |  | Transcription factors | pMMR | 0.02 |
|  | **Membrane Transport** |  |  |  |
|  |  | Secretion system | pMMR | 0.02 |
|  | **Signaling Molecules and Interaction** |  |  |  |
|  |  | G protein-coupled receptors | dMMR | ＜0.001 |
|  | **Signal Transduction** |  |  |  |
|  |  | Calcium signaling pathway | dMMR | 0.008 |
|  |  | Notch signaling pathway | dMMR | 0.03 |
|  |  | Wnt signaling pathway | dMMR | 0.03 |
|  | **Amino Acid Metabolism** |  |  |  |
|  |  | Lysine degradation | pMMR | ＜0.001 |
|  |  | Tryptophan metabolism | pMMR | 0.001 |
|  |  | Tyrosine metabolism | pMMR | 0.009 |
|  |  | Valine, leucine and isoleucine degradation | pMMR | 0.002 |
|  | **Carbohydrate Metabolism** |  |  |  |
|  |  | Butanoate metabolism | pMMR | 0.002 |
|  |  | Galactose metabolism | dMMR | 0.02 |
|  |  | Glyoxylate and dicarboxylate metabolism | pMMR | 0.03 |
|  |  | Propanoate metabolism | pMMR | 0.001 |
|  | **Energy Metabolism** |  |  |  |
|  |  | Methane metabolism | pMMR | 0.04 |
|  |  | Nitrogen metabolism | pMMR | 0.01 |
|  |  | Photosynthesis - antenna proteins | dMMR | 0.007 |
|  | **Glycan Biosynthesis and Metabolism** |  |  |  |
|  |  | Glycosaminoglycan degradation | dMMR | ＜0.001 |
|  |  | Glycosphingolipid biosynthesis - ganglio series | dMMR | ＜0.001 |
|  |  | Glycosphingolipid biosynthesis - globo series | dMMR | ＜0.001 |
|  |  | Glycosphingolipid biosynthesis - lacto and neolacto series | pMMR | ＜0.001 |
|  |  | Lipopolysaccharide biosynthesis | pMMR | ＜0.001 |
|  |  | Lipopolysaccharide biosynthesis proteins | pMMR | ＜0.001 |
|  |  | N-Glycan biosynthesis | dMMR | ＜0.001 |
|  |  | Other glycan degradation | dMMR | ＜0.001 |
|  |  | Various types of N-glycan biosynthesis | dMMR | ＜0.001 |
|  | **Lipid Metabolism** |  |  |  |
|  |  | alpha-Linolenic acid metabolism | dMMR | 0.04 |
|  |  | Arachidonic acid metabolism | pMMR | 0.02 |
|  |  | Biosynthesis of unsaturated fatty acids | pMMR | ＜0.001 |
|  |  | Ether lipid metabolism | pMMR | 0.03 |
|  |  | Fatty acid elongation in mitochondria | pMMR | ＜0.001 |
|  |  | Fatty acid metabolism | pMMR | ＜0.001 |
|  |  | Linoleic acid metabolism | pMMR | ＜0.001 |
|  |  | Primary bile acid biosynthesis | pMMR | ＜0.001 |
|  |  | Sphingolipid metabolism | dMMR | 0.02 |
|  |  | Sphingolipid metabolism | dMMR | 0.01 |
|  |  | Steroid biosynthesis | pMMR | 0.03 |
|  |  | Steroid hormone biosynthesis | dMMR | ＜0.001 |
|  |  | Synthesis and degradation of ketone bodies | pMMR | 0.01 |
|  | **Metabolism of Cofactors and Vitamins** |  |  |  |
|  |  | Biotin metabolism | pMMR | 0.01 |
|  | **Metabolism of Other Amino Acids** |  |  |  |
|  |  | beta-Alanine metabolism | pMMR | ＜0.001 |
|  |  | D-Arginine and D-ornithine metabolism | pMMR | 0.001 |
|  |  | Phosphonate and phosphinate metabolism | dMMR | 0.002 |
|  | | Taurine and hypotaurine metabolism | pMMR | 0.03 |
